# Supplementary material for: In vivo single-cell RNA metabolic labeling resolves early transcriptional responders in the regenerating zebrafish heart
Source: Nat Commun. 2026 May 5;17:4073. doi: 10.1038/s41467-026-72781-2 (PMC13144409; doi:10.1038/s41467-026-72781-2)
Supplement: Supplementary file 1 — Supplementary Information [file 41467_2026_72781_MOESM1_ESM.pdf]

Supplement Content:

1. Supplementary Figures + Legends
2. Mathematical Supplement

Supplementary Fig. 1

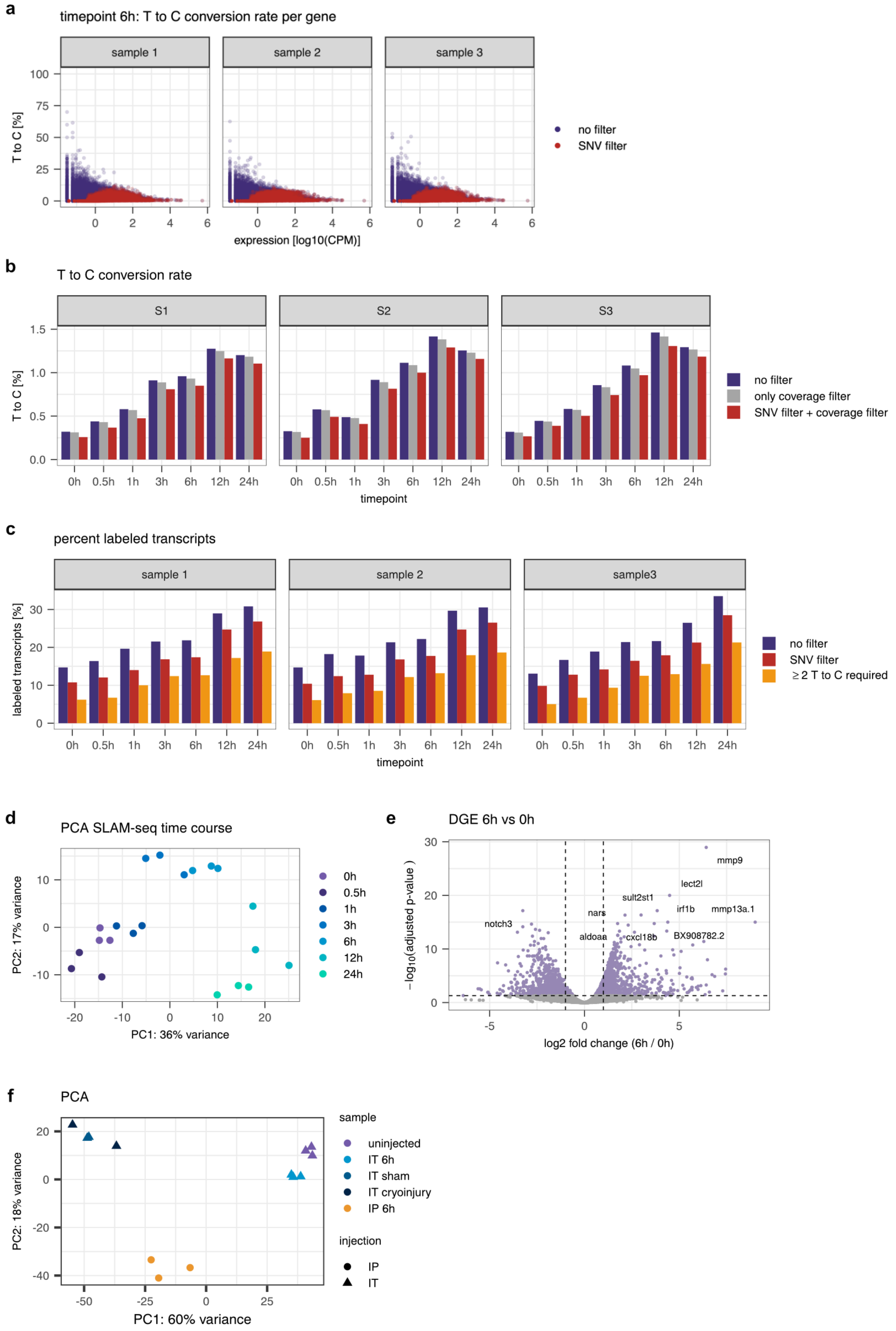

Supplementary Figure 1 Effects of SNVs and a stricter conversion filter on labeling rates. a, Bulk SLAM-seq T to C conversion rates per gene as a function of expression of the 6h 4sU labeling timepoint per biological replicate comparing labeling rates without filtering and with SNV filtering. Each dot represents one gene. **b,** Bulk SLAM-seq T to C conversion rates across time, comparing labeling rates across time without filtering (at least 1 T to C conversion required to call a transcript labeled), with SNV filtering including a coverage filter, as well as only the coverage filter without SNV filtering. S1-S3 … Sample 1-3 **c,** Percentage of labeled transcripts (UMI-based) in SLAM-seq time course, comparing labeling across time without filter (at least 1 T to C conversion required to call a transcript labeled), with SNV filter and with at least 2 conversions required to classify a transcript as labeled. **d,** PCA of bulk SLAM-seq time course data based on total, i.e. labeled + unlabeled counts. **e,** Volcano plot of differential gene expression of 6 h vs. 0 h (uninjected) time course datapoints. **f,** PCA of uninjected, IT-injected 6 h, IT injected 6 hps (sham), IT-injected 6 hpci (cryoinjury) and IP injected 6 h samples based on total, i.e. labeled + unlabeled, reads.

Supplementary Fig. 2

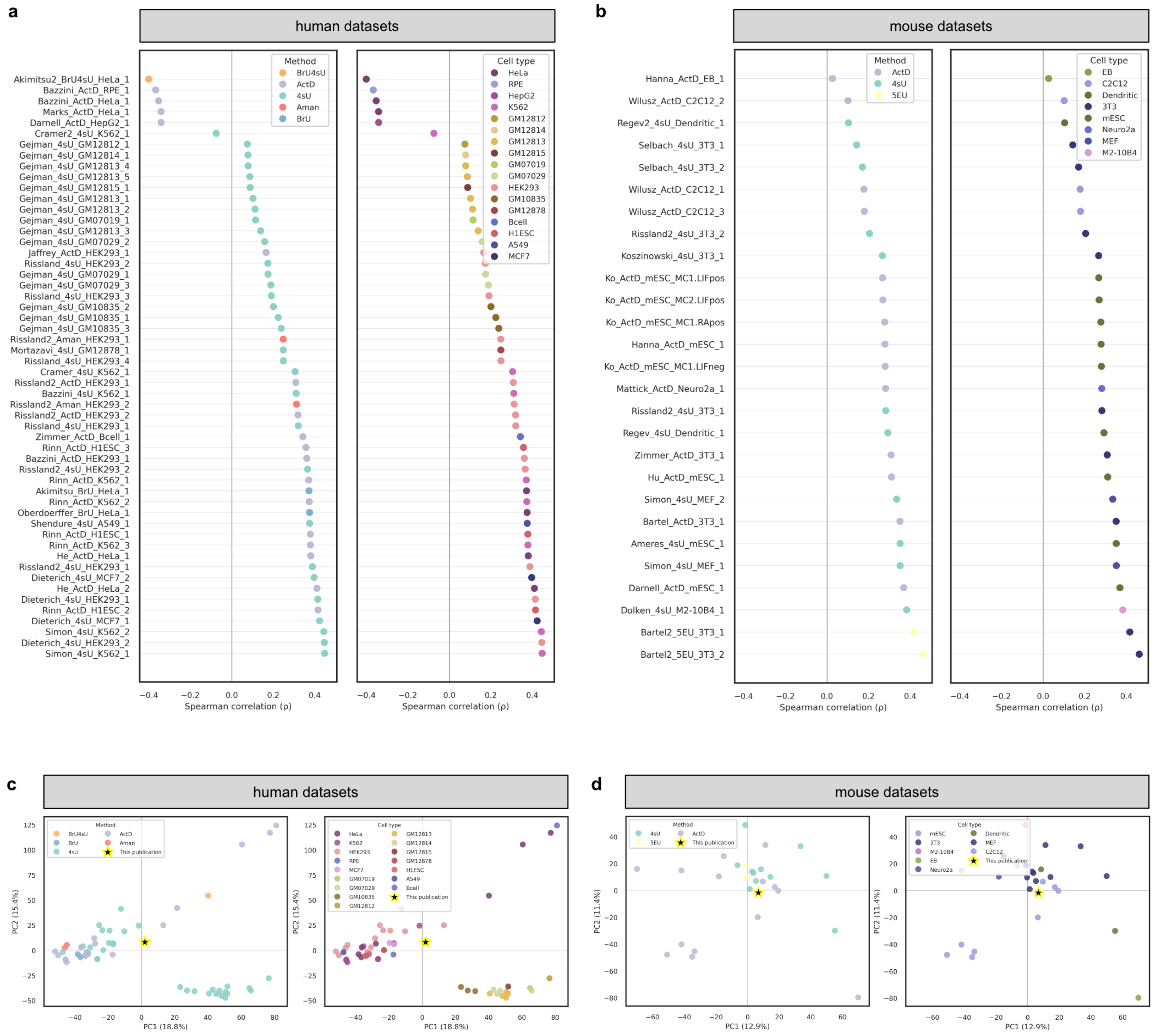

Supplementary Figure 2 Cross-species comparison of mRNA stability measurements. **a,** Spearman correlations (ρ) between zebrafish gene-level half-life z-scores and published half-life z-scores from Agarwal & Kelley 2022 across human datasets. Each point represents one dataset (computed across orthologous genes quantified in both zebrafish and the respective human dataset) and datasets are ordered by correlation value. Points are colored by experimental labeling or transcriptional inhibition method (left; ActD, 4sU, BrU4sU, BrU, Aman or 5EU) or by originating cell type (right; HEK293, HeLa, K562, RPE, H1ESC, A549, MCF7, HepG2, GM lymphoblastoid lines, B cell). The vertical grey line indicates $\rho = 0$. **b,** Same analysis as in a for mouse datasets, with points colored by experimental method (left) or by cell type (right; mESC, 3T3, MEF, EB, Neuro2a, Dendritic, C2C12 or M2-10B4). **c,** Principal component analysis (PCA) of gene-level half-life z-scores across human + zebrafish datasets. A gene × dataset matrix of orthologous genes was constructed, retaining genes quantified in ⩾50% of the datasets and imputing remaining missing values with the gene-wise mean. Datasets, including the zebrafish decay vector, were z-scored and projected into PCA space; points are colored by experimental method (left) or by cell type (right). The zebrafish dataset from this study is highlighted with a star; axes indicate PC1 and PC2 with the corresponding explained variance. **d,** Same PCA analysis as in c for mouse + zebrafish datasets, with points colored by experimental method (left) or by cell type (right).

Supplementary Fig. 3

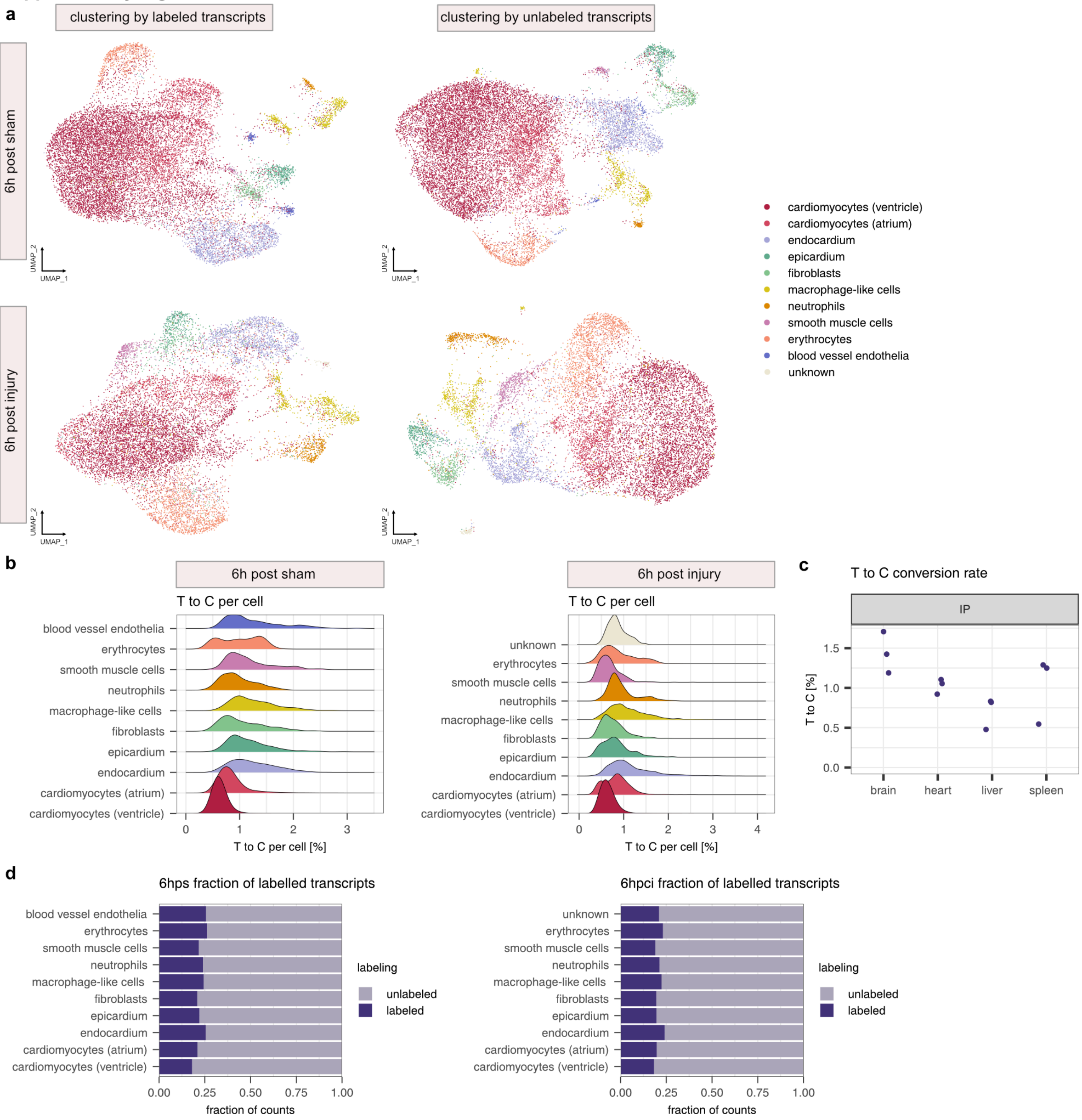

Supplementary Figure 3 Characterization of scSLAM-seq data. a, UMAPs of 6 hps (top) and 6 hpci (bottom) scSLAM-seq data, clustered by labeled transcripts only (left) and unlabeled transcripts only (right). **b,** T to C conversion rate per cell of scSLAM-seq 6 hps (left) and 6 hpci (right) datasets. Each transcript within a cell contributes equally to the calculated conversion rate per cell, and are therefore more strongly influenced by highly abundant transcripts and housekeeping genes. **c,** T to C conversion rates of bulk SLAM-seq of different organs IP injected with 200 mM 4sU after 6 h labeling. Sample size: n = 3, brain, heart, liver and spleen were dissected from each zebrafish. **d,** Fraction of labeled transcripts per cell type of scSLAM-seq 6 hps (left) and 6 hpci (right) datasets.

Supplementary Fig. 4

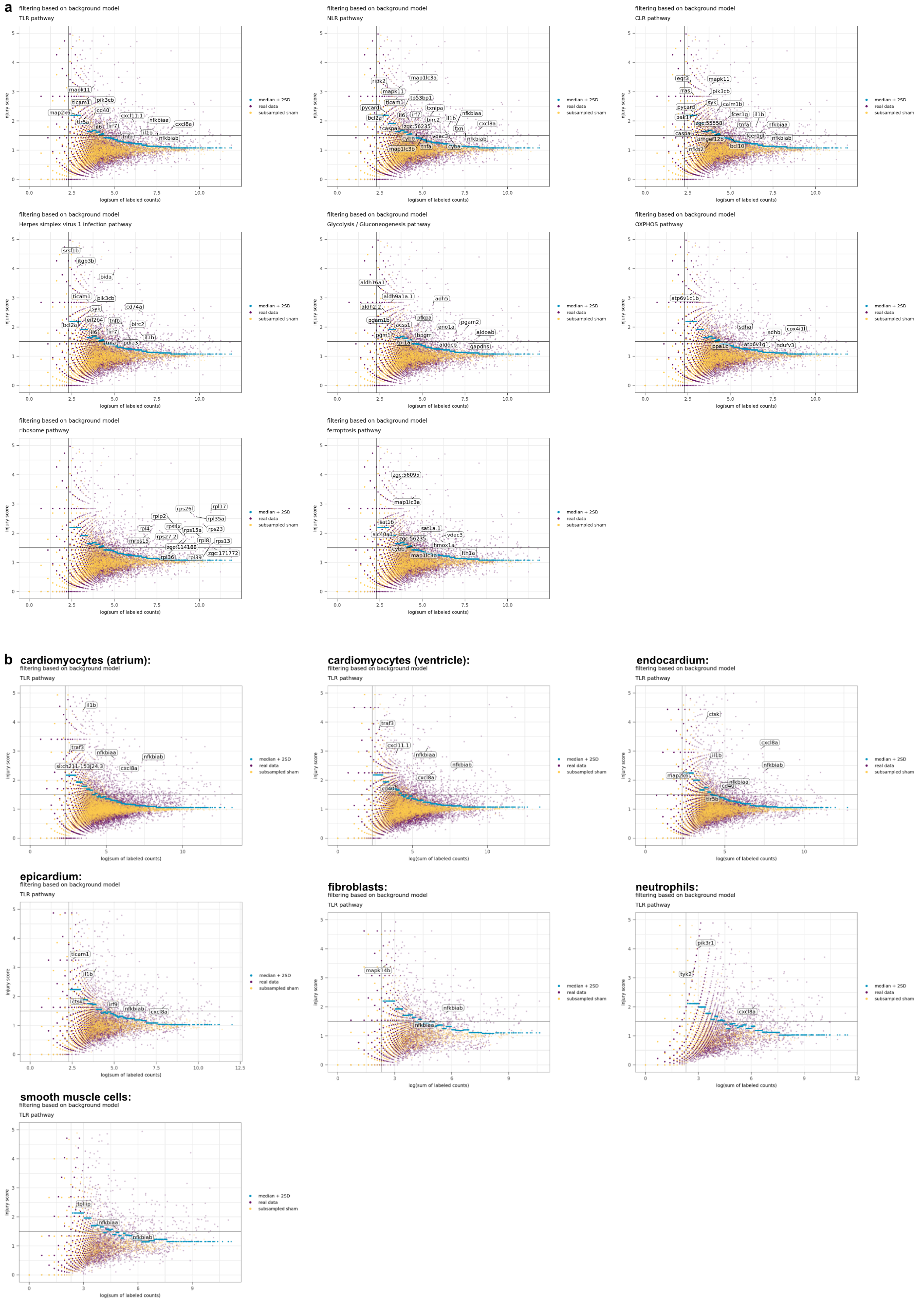

Supplementary Figure 4 Pathway-specific upregulation of genes in the scSLAM-seq injury response

a, scSLAM-seq background model plots (see mathematical supplement) with pathway-specific genes which are identified as injury-responsive in macrophage-like cells. **b,** scSLAM-seq background model plots with TLR signaling pathway-specific genes which are identified as injury-responsive in all other cell types.

Supplementary Fig. 5

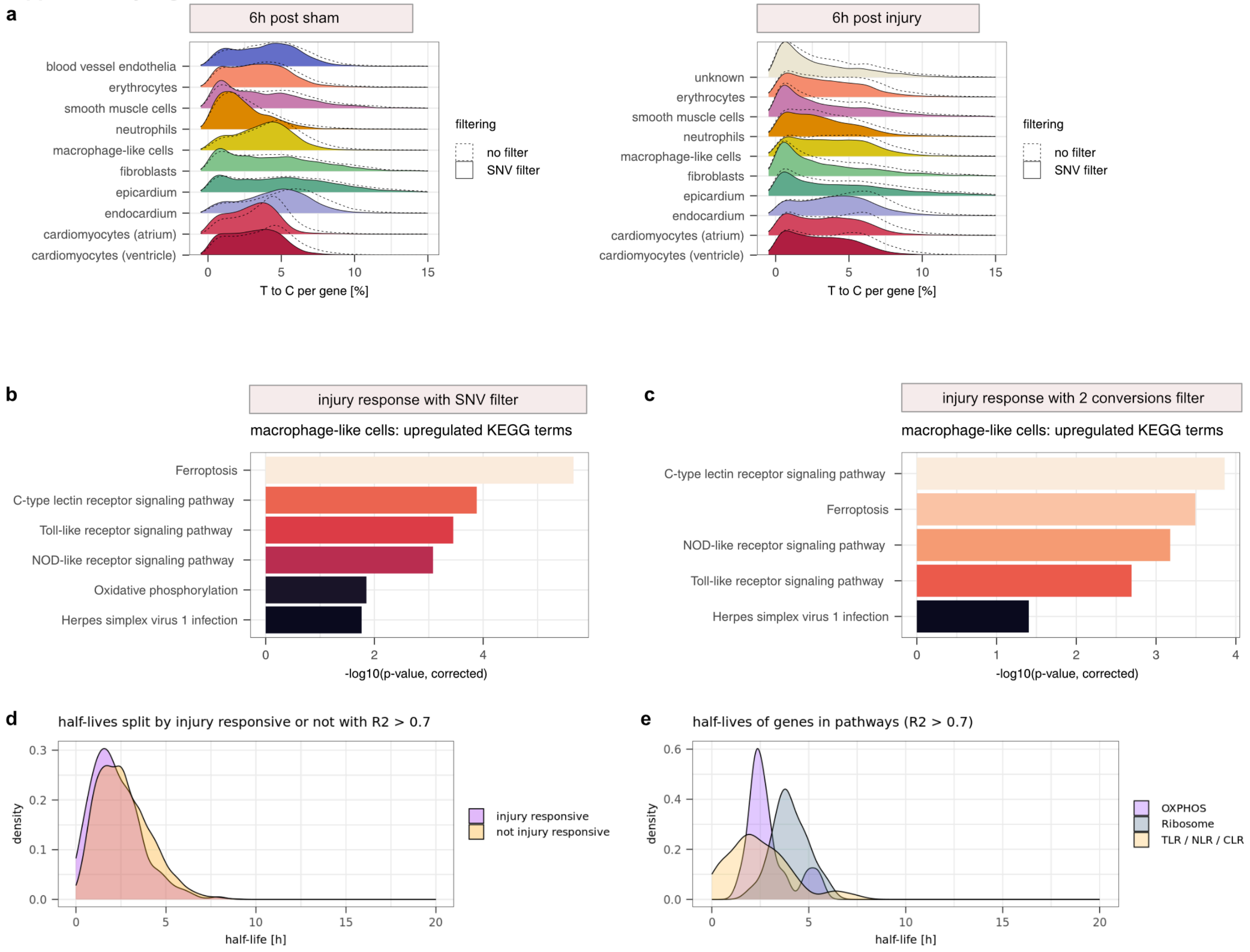

Supplementary Figure 5 Effect of SNV and a stricter conversion filter on the measured injury response in scSLAM-seq data and link of RNA half-lives to injury response. a, T to C conversion per gene by cell type of scSLAM-seq 6 hps (left) and 6 hpci (right). Filled areas indicate T to C conversions per gene after SNV filtering, while the dashed line shows the distribution without SNV filtering. **b,** Upregulated KEGG terms of injury responsive genes in macrophage-like cells after SNV filtering. **c,** Upregulated KEGG terms of injury responsive genes in macrophage-like cells requiring at least two T to C conversions to call a transcript labeled. **d,** Half-lives of injury-responsive versus expressed, but not injury-responsive genes in macrophage-like cells with a fit R^2>0.7. **e,** Half-lives of all genes of the OXPHOS, Ribosome and pro-inflammatory (TLR, NLR and CLR) KEGG pathways (irrespective of scSLAM-seq injury response) with a fit R^2>0.7.

Supplementary Fig. 6

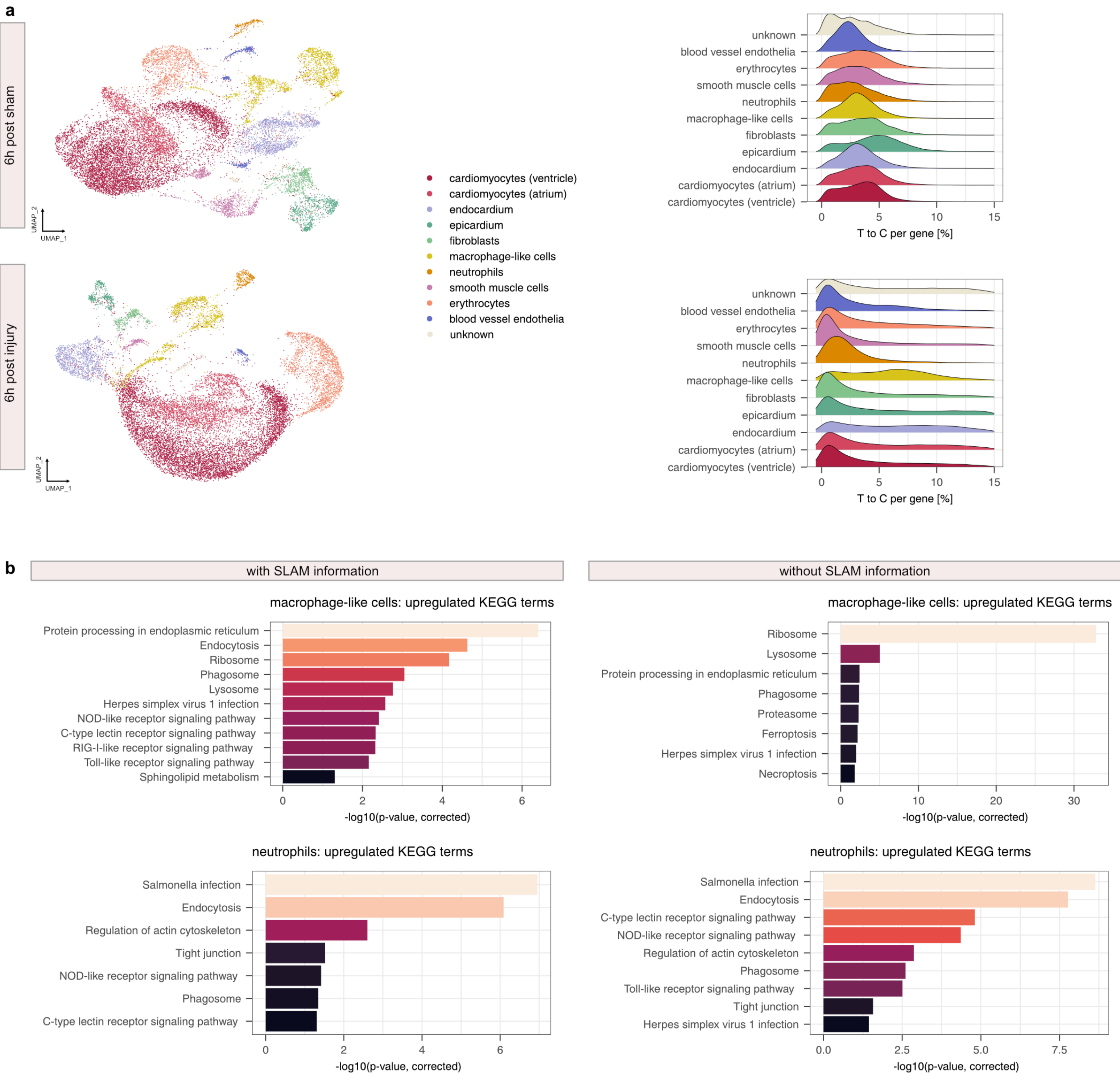

Supplementary Figure 6 scSLAM-seq replicate data and injury response. a, UMAPs (left) of 6 hps (top) and 6 hpci (bottom) scSLAM-seq replicates and their respective T to C conversion rate distribution per gene by cell type (right). Sample size: 6 hps one 10X run of a pool of 4 biological replicates, 6 hpci one 10X run of a pool of 5 biological replicates. **b,** Upregulated KEGG terms of injury responsive genes in macrophage-like cells and neutrophils using SLAM information (left) and no SLAM information i.e. the transcriptome consisting of labeled as well as unlabeled RNA (right).

Supplementary Fig. 7

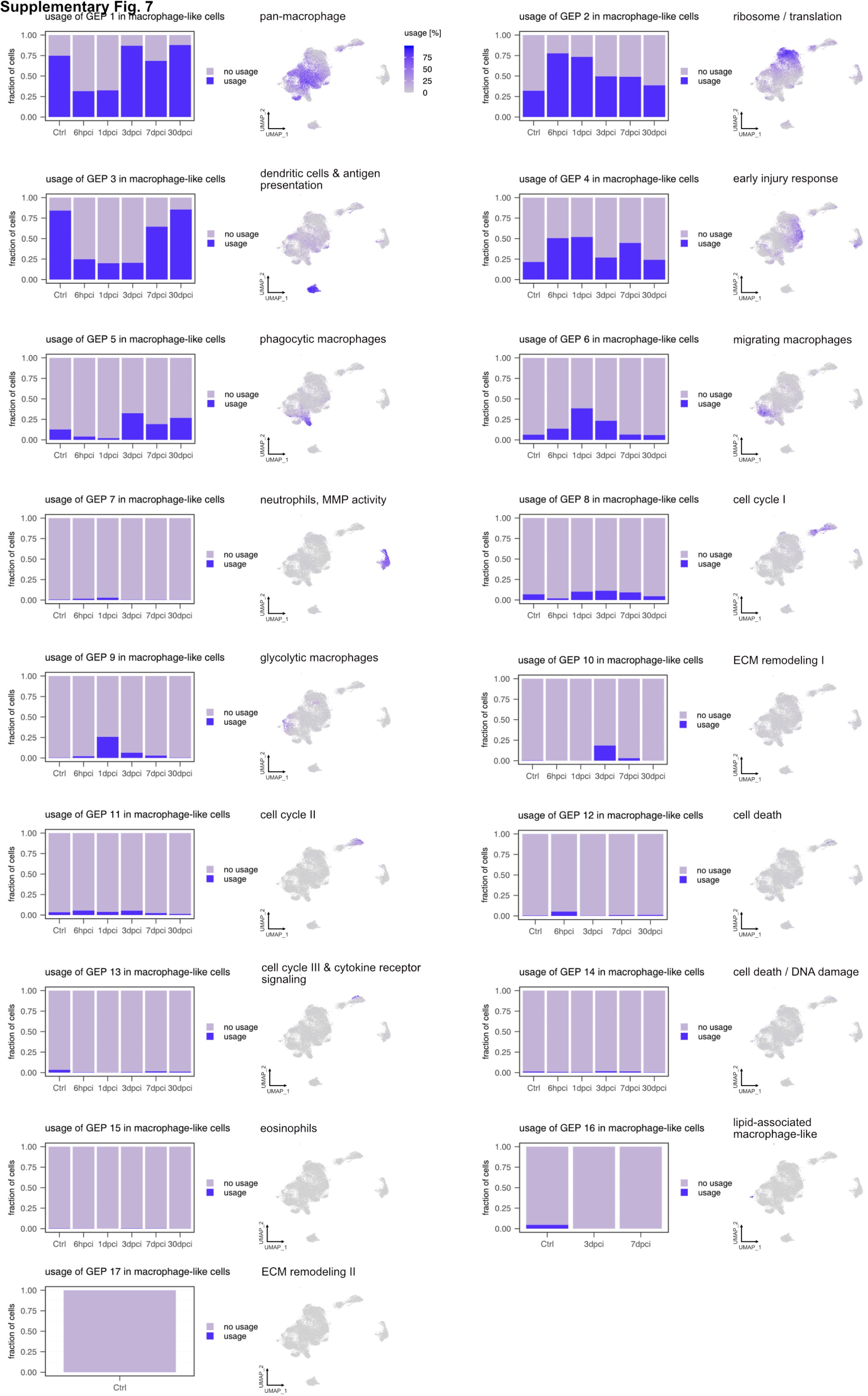

Supplementary Figure 7 Gene expression programs identified with cNMF. Gene expression programs of myeloid cells identified with cNMF. Bar plots indicate the fraction of macrophage-like cells that use a GEP (required cutoff: 10% usage per cell) per time point. UMAPs indicate usage in percent of the gene expression program.

Supplementary Fig. 8

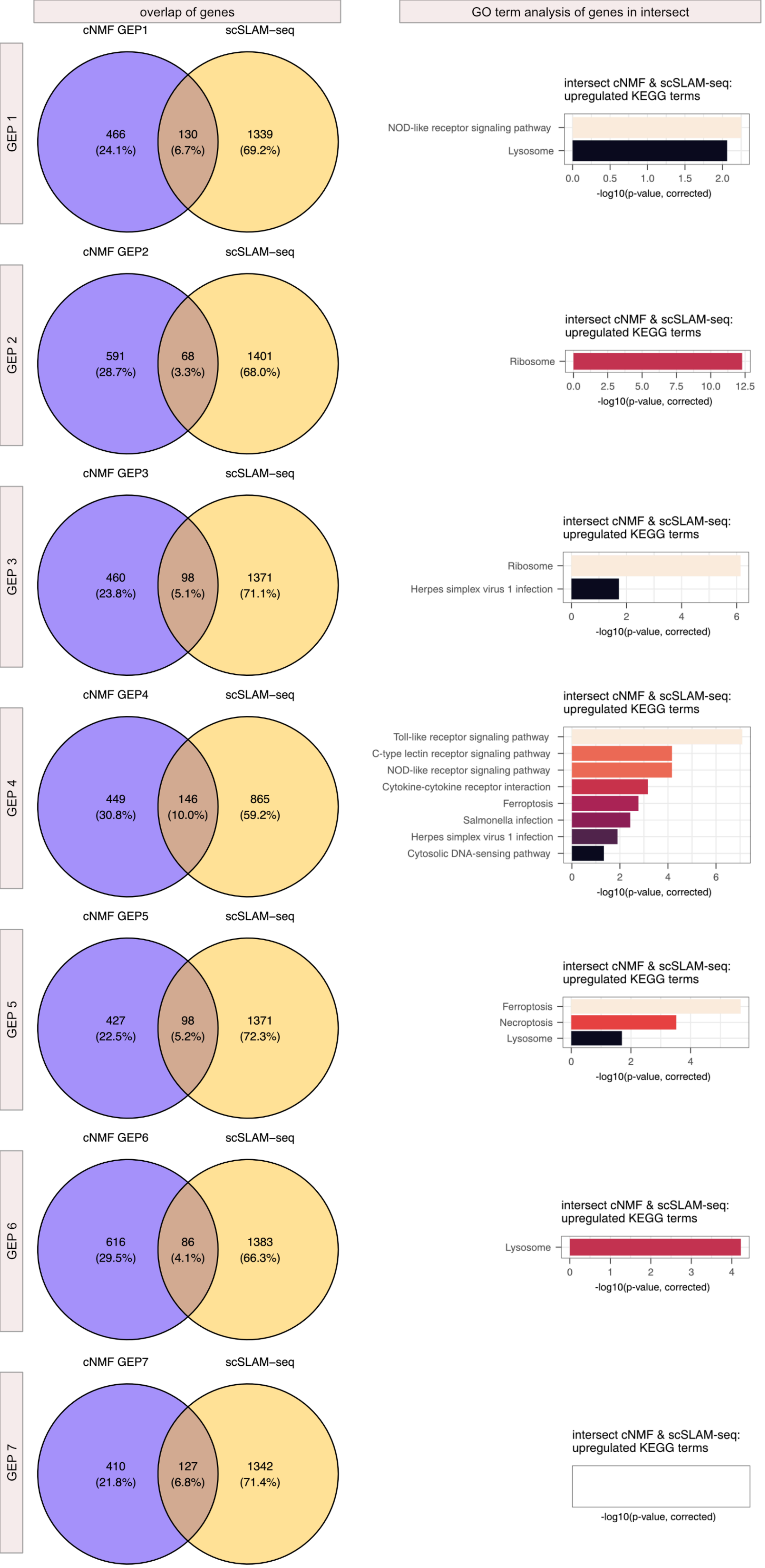

Supplementary Figure 8 Overlap of cNMF and scSLAM-seq injury responsive genes. Overlap of genes upregulated in gene expression programs and genes identified as injury responsive from scSLAM-seq data (left) and upregulated KEGG terms of the intersect of genes (right).

Supplementary Fig. 9

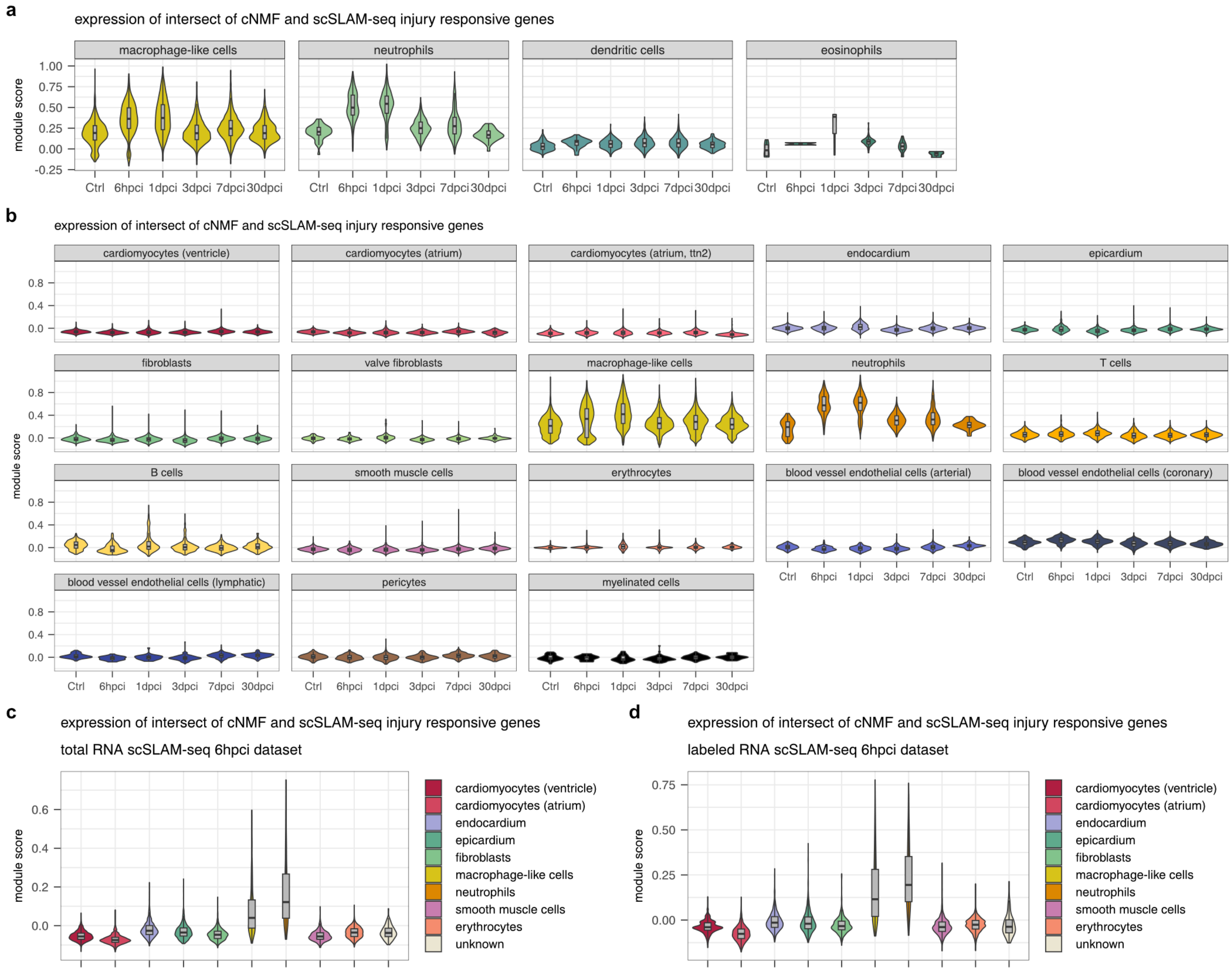

Supplementary Figure 9 Scoring of injury-responsive genes. a, Seurat module scores of the intersect of genes from injury-responsive genes identified in macrophage-like cells with scSLAM-seq and the injury-responsive GEP in the myeloid subcluster of the heart atlas. Clusters were grouped into cell types (macrophage-like cells, neutrophils, dendritic cells, eosinophils). **b,** Seurat module scores of the intersect of genes in all cell types of the heart atlas. **c,** Seurat module score of the intersect of genes in the total RNA (i.e. labeled and unlabeled) of the 6 hpci scSLAM-seq sample. **d,** Seurat module score of the intersect of genes in the labeled RNA of the 6 hpci scSLAM-seq sample.

Supplementary Fig. 10

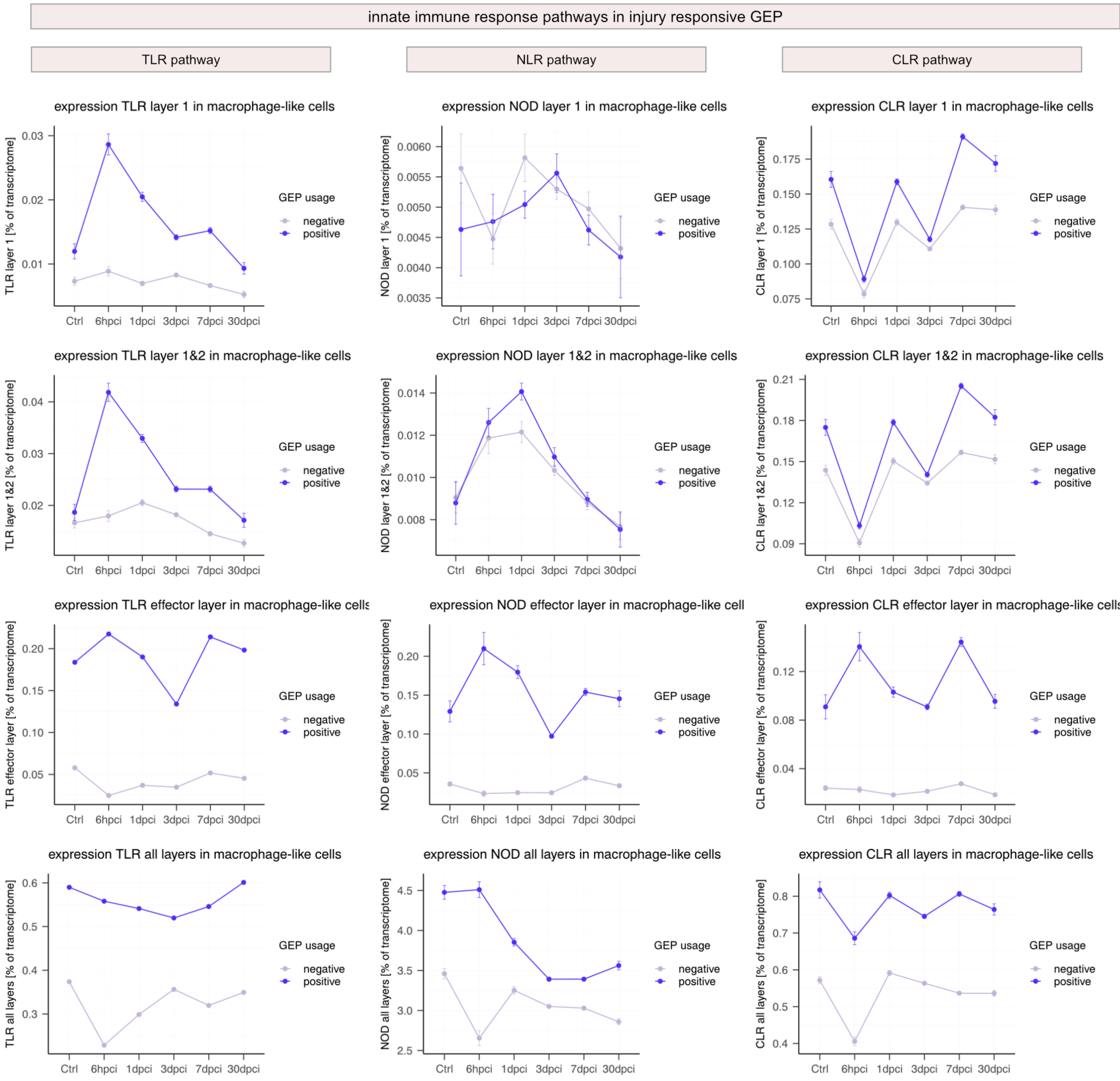

Supplementary Figure 10 Innate immune response pathway layer expression in macrophage-like cells. Expression of TLR, NLR and CLR pathway layers in macrophage-like cells as percent transcriptome across timepoints and split by cells using or not using the injury responsive GEP (required usage cut-off: 10%). “All layers” refers to all molecules in the pathway. Depicted is the average expression ± SEM.

Supplementary Fig. 11

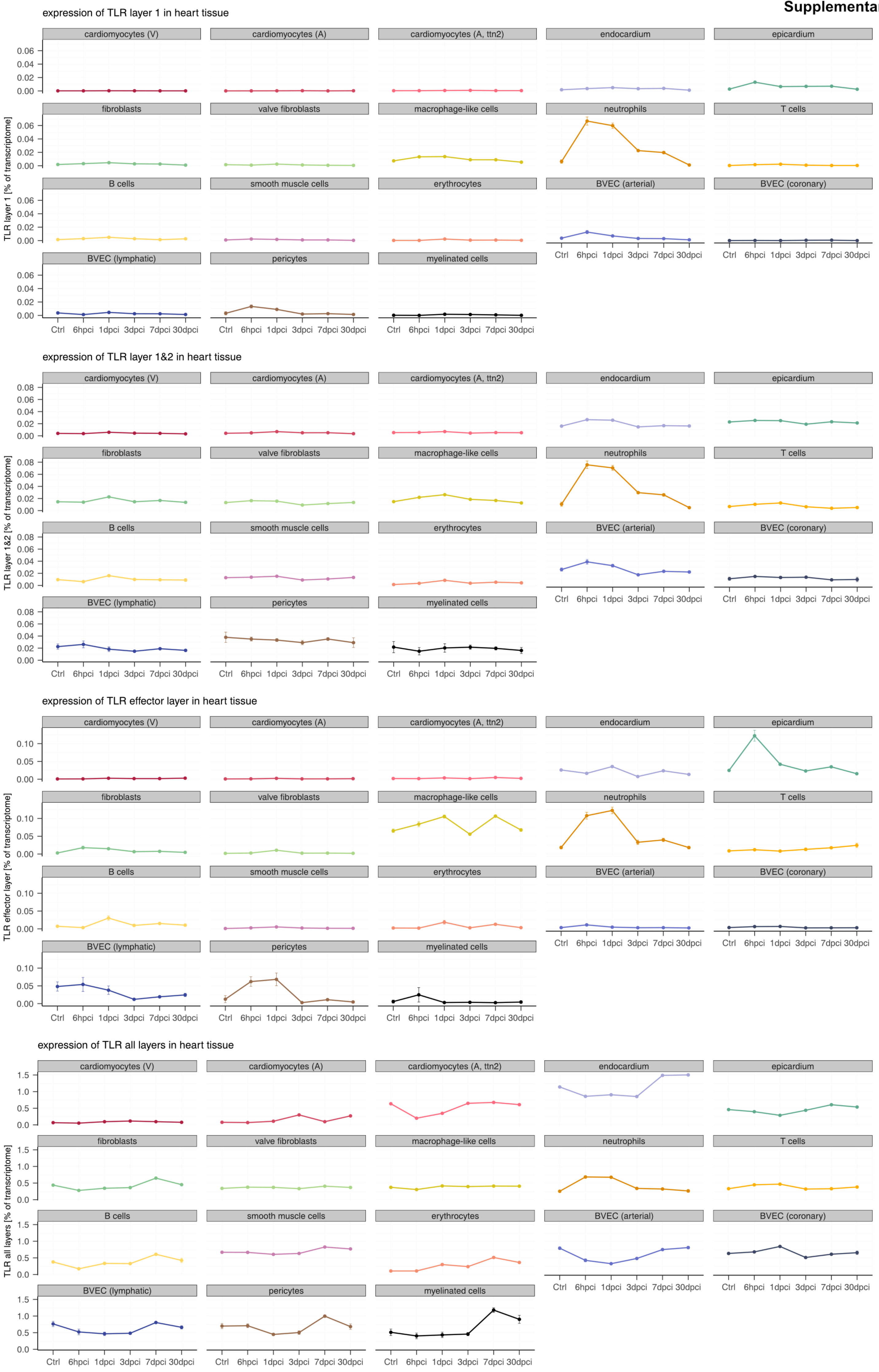

Supplementary Figure 11 Innate immune response pathway layer expression across all cell types in the zebrafish heart. Expression of TLR pathway layers across all cell types of the heart scRNA-seq atlas by time point. Depicted is the average expression ± SEM.

Supplementary Fig. 12

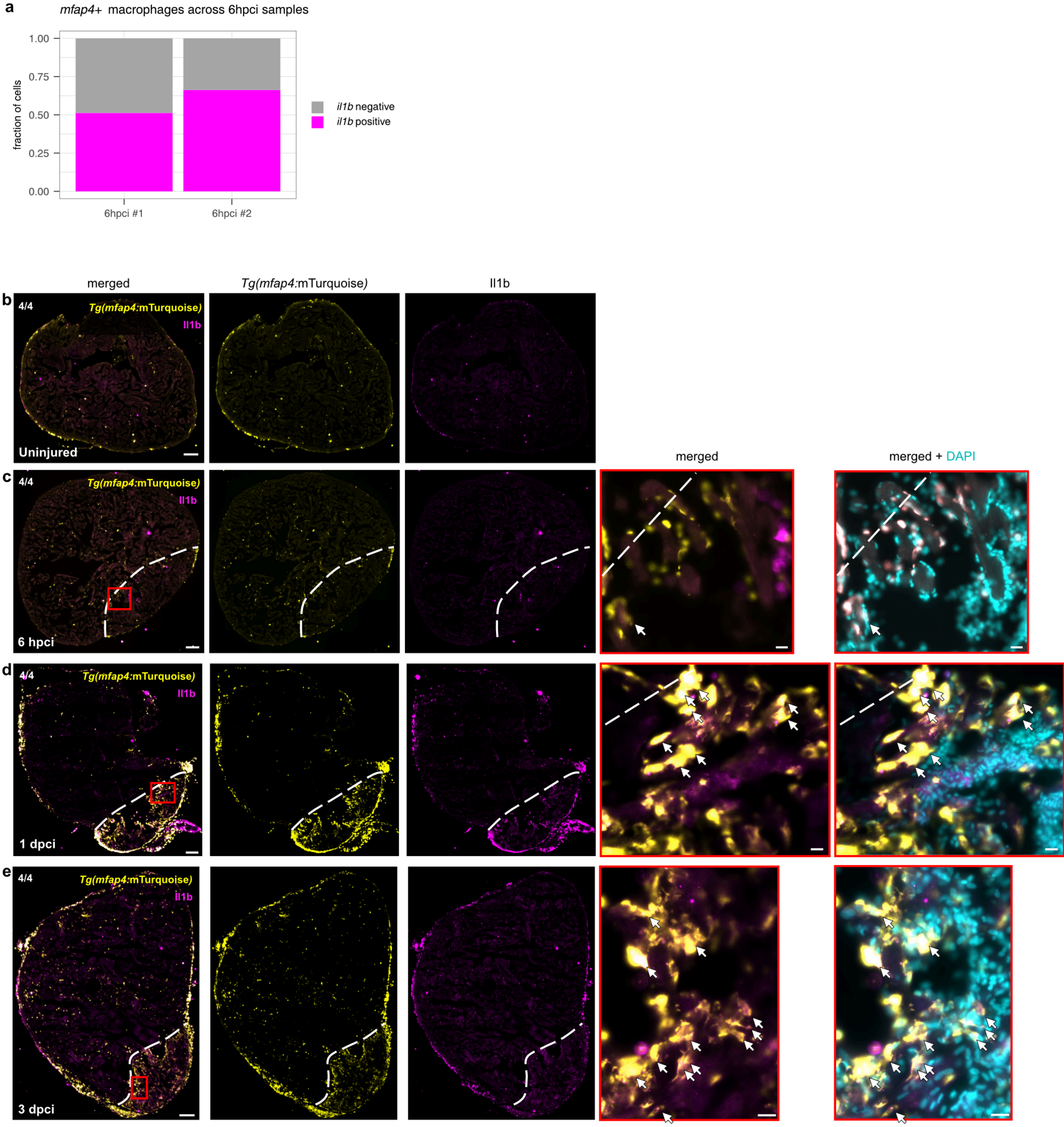

Supplementary Figure 12 Pro-inflammatory macrophages are recruited into the injured area after cardiac cryoinjury.

a, Quantification of *mfap4.1*+ (gray) and *mfap4.1*+ *il1b*+ (pink) positive cells from HCR on 6 hpci heart cryosections. Number of sections imaged in 6 hpci #1: 6, 6 hpci #2: 3. **b,** Representative immunostaining images of pro-inflammatory macrophages (Mfap4^{+} Il1b^{+}) in heart section from uninjured *Tg(mfap4:mTurquoise)* zebrafish; n = 4. **c-e,** Representative immunostaining images of pro-inflammatory macrophages (*mfap4:*mTurquoise^{+} Il1b^{+}) in heart sections from cryoinjured *Tg(mfap4:mTurquoise)* zebrafish at 6 hpci (n = 4) (**c**), 1 dpci (n = 4) (**d**), and 3 dpci (n = 4) (**e**). Magnified views of red-boxed regions are shown in the right panels. White arrows point to pro-inflammatory macrophages (*mfap4:*mTurquoise^{+} Il1b^{+}). White dashed lines outline the injured area. Scale bars: 100 µm and 10 µm (magnified images).

Supplementary Fig. 13

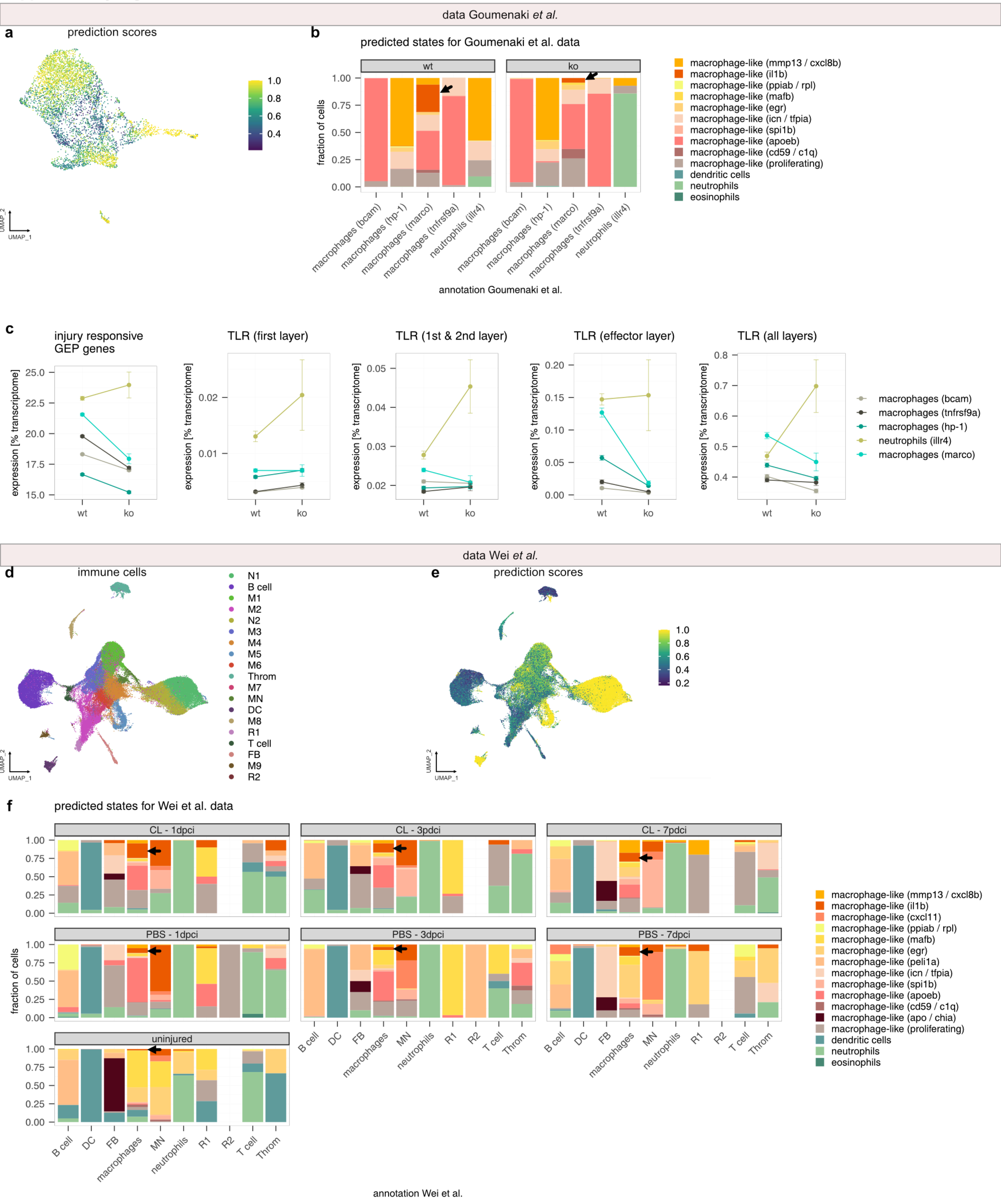

Supplementary Figure 13 Label transfer and analysis of the injury responsive cell state in published datasets. a, Prediction scores of the label transfer from our myeloid data onto the data of Goumenaki *et al.* **b,** Results of label transfer from our myeloid data onto the data of Goumenaki *et al.*[15], split by wt (*myd88*$^{+/+}$) and ko (*myd88*$^{-/-}$) and published annotations of Goumenaki *et al.*[15] Depicted is the fraction of cells per condition assigned to clusters from our data. Black arrows point towards the fraction of cells that were assigned to the *macrophage-like cells (il1b)* cluster with label transfer. **c,** Expression of the top genes of the injury responsive GEP as well as TLR pathway layers in Goumenaki *et al*.[15] myeloid cells split by wt (*myd88*$^{+/+}$) and ko (*myd88*$^{-/-}$) and colored by the published annotations. Depicted are the average expression per group ± SEM. **d,** UMAP of immune cells from Wei *et al.*[16] (using published cluster annotations and UMAP embeddings). **e,** Prediction scores of the label transfer from our myeloid data onto the data of Wei *et al.*[16] **f,** Results of label transfer from our myeloid data onto the data of Wei *et al.*[16], split by condition (clodronate liposomes as “CL” or PBS injection as well as time point) as well as published annotations of Wei *et al*.[16] Depicted is the fraction of cells per condition assigned to clusters from our data. In this analysis, we summarized M1 through M9 clusters to “macrophages”. Black arrows point towards the fraction of cells in the macrophage cluster that were assigned to the *macrophage-like cells (il1b)* cluster with label transfer.

Supplementary Fig. 14

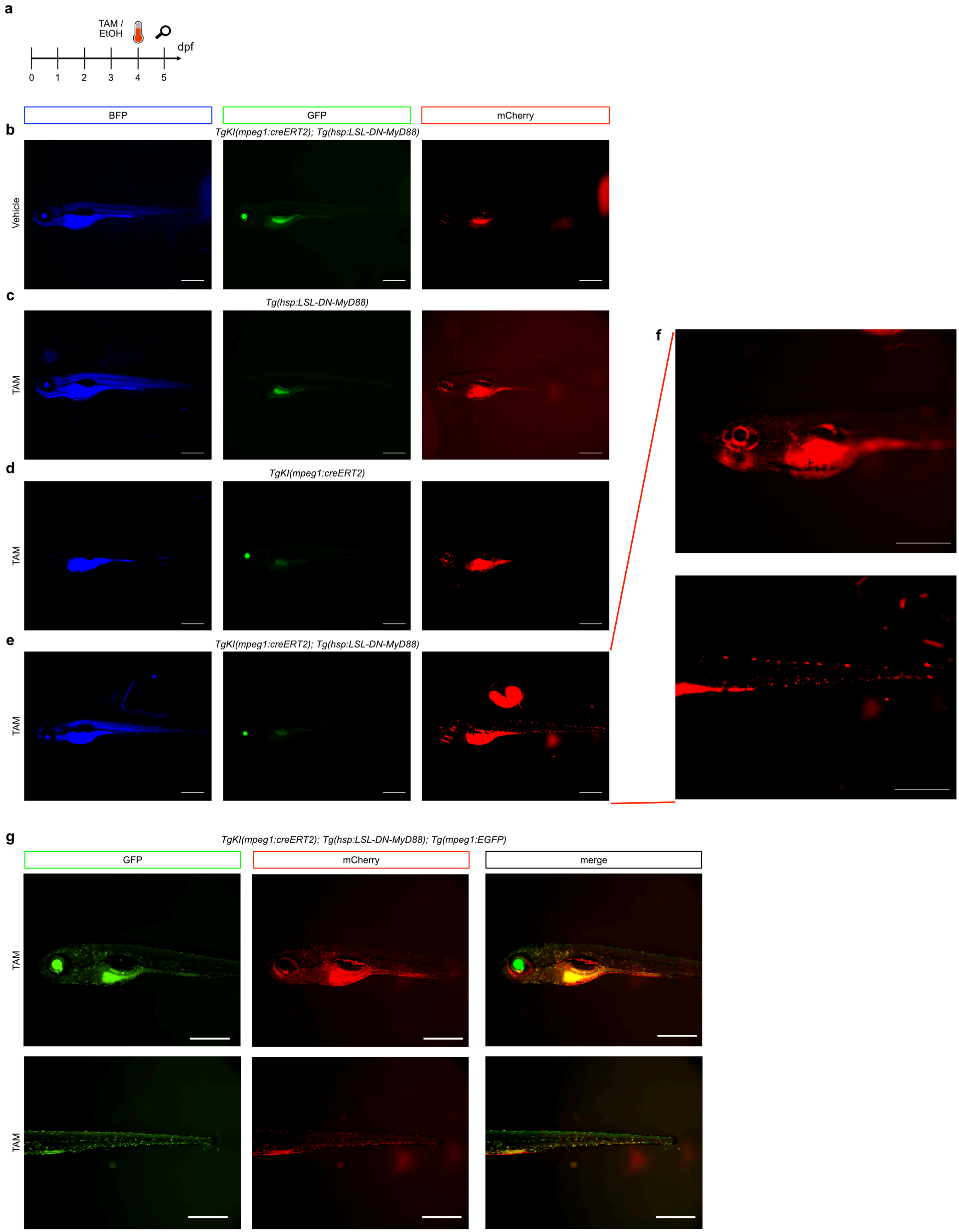

Supplementary Figure 14 Testing recombination in *TgKI(mpeg1:CreERT2);Tg(hsp:LSL-DN-MyD88)* zebrafish larvae. a, Experimental design to validate macrophage-specific induction of DN-MyD88 expression following TAM and heat-shock treatment (thermometer) in zebrafish larvae. **b,** Representative fluorescence images of *TgKI(mpeg1:CreERT2);Tg(hsp:LSL-DN-MyD88)* larvae treated with vehicle at 5dpf, showing no recombination. **c,** Representative fluorescence images of *Tg(hsp:LSL-DN-MyD88)* larvae treated with TAM at 5 dpf, lacking CreER expression, showing no recombination. **d,** Representative fluorescence images of *TgKI(mpeg1:CreERT2)* larvae treated with TAM at 5 dpf, lacking the DN-MyD88 construct, showing no overexpression. **e,** Representative fluorescence images of *TgKI(mpeg1:CreERT2);Tg(hsp:LSL-DN-MyD88)* larvae treated with TAM at 5 dpf, showing mCherry expression in *mpeg1*+ macrophages, indicating successful Cre-mediated recombination. **f,** Zoom-in images of (e). **g**, Representative fluorescence images of *TgKI(mpeg1:CreERT2);Tg(hsp:LSL-DN-MyD88); Tg (mpeg1:eGFP)* larvae treated with TAM at 5 dpf, showing mCherry expression in *mpeg1*$^+$ macrophages, indicating successful Cre-mediated recombination in *mpeg1*$^+$ macrophages. Scale bars: 500 µm. Experiments were repeated 3 times with 10 larvae per experimental condition.

Supplementary Fig. 15

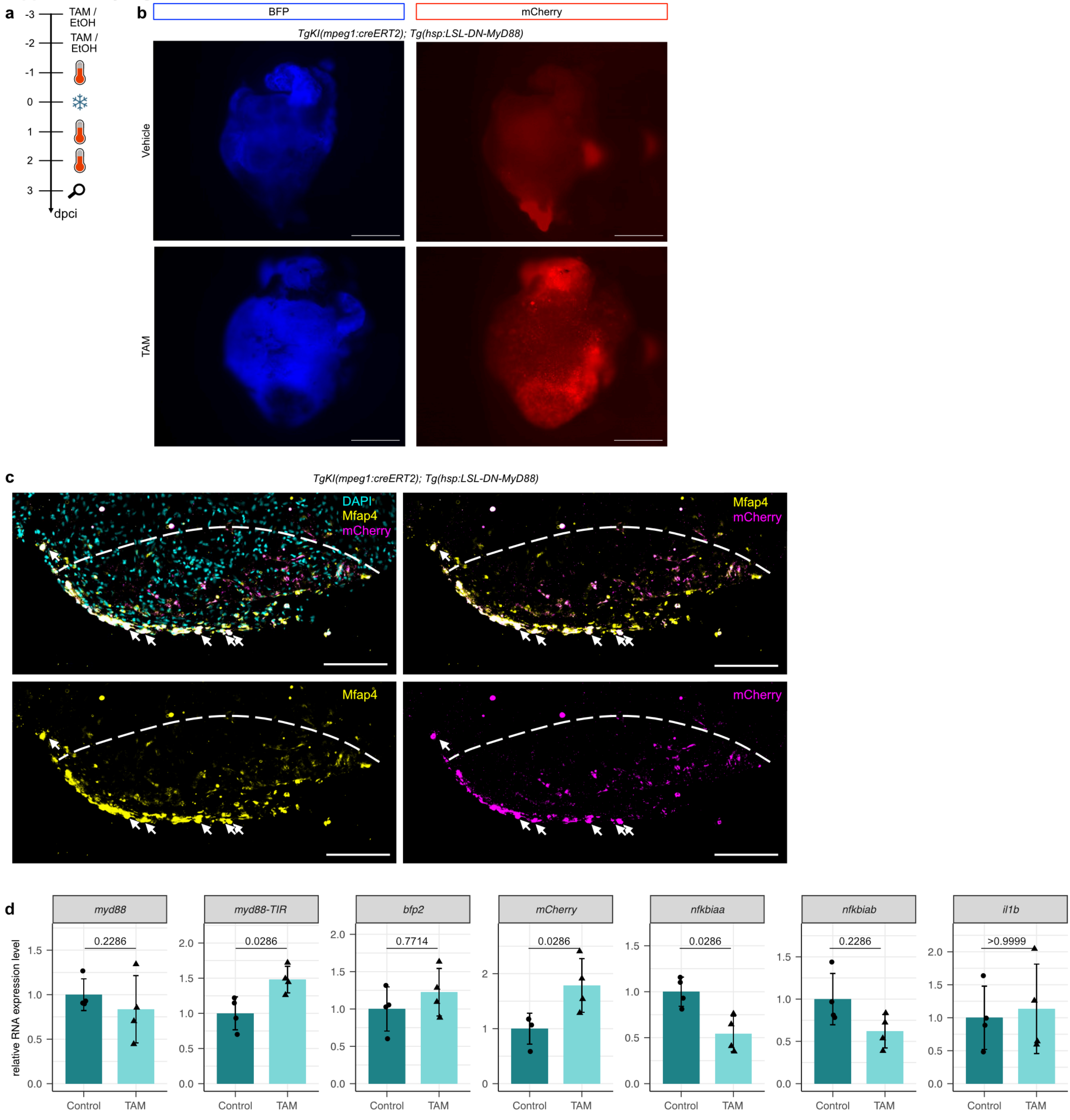

Supplementary Fig. 15 Testing recombination in *TgKI(mpeg1:CreERT2);Tg(hsp:LSL-DN-MyD88)* adult zebrafish.

a, Experimental design to validate macrophage-specific induction of DN-MyD88 expression following TAM and heat-shock treatment (thermometer) in cryoinjured zebrafish. **b,** Representative fluorescence images of *TgKI(mpeg1:CreERT2);Tg(hsp:LSL-DN-MyD88)* zebrafish treated with vehicle or TAM at 3 dpci. Sample size: n=4 (for both TAM and vehicle control treated). Scale bars: 500 μm. **c**, Representative immunostaining images for Mfap4 (macrophage, yellow) and mCherry (recombined signal, magenta) in heart sections from cryoinjured *TgKI(mpeg1:CreERT2);Tg(hsp:LSL-DN-MyD88)* zebrafish treated with TAM at 3 dpci. White dashed lines outline the injured area. White arrows point to the double positive cells (Mfap4$^+$ and mCherry$^+$). Sample size: n=4. Scale bars: 100 μm. **d,** Relative expression levels of *myd88* (endogenous), *myd88-TIR* (endogenous + dn-myd88), *bfp2*, *mCherry*, *nfkbiaa*, *nfkbiab*, and *il1b* in whole ventricle from cryoinjured *TgKI(mpeg1:CreERT2);Tg(hsp:LSL-DN-MyD88)* zebrafish treated with vehicle or TAM at 3 dpci. Dots represent individual ventricles; data shown as mean ± s.d.; statistical test: Student´s t-test. **a** was partially created in BioRender. Mintcheva, J. (2026) https://BioRender.com/w02lqzq

Supplementary Fig. 16

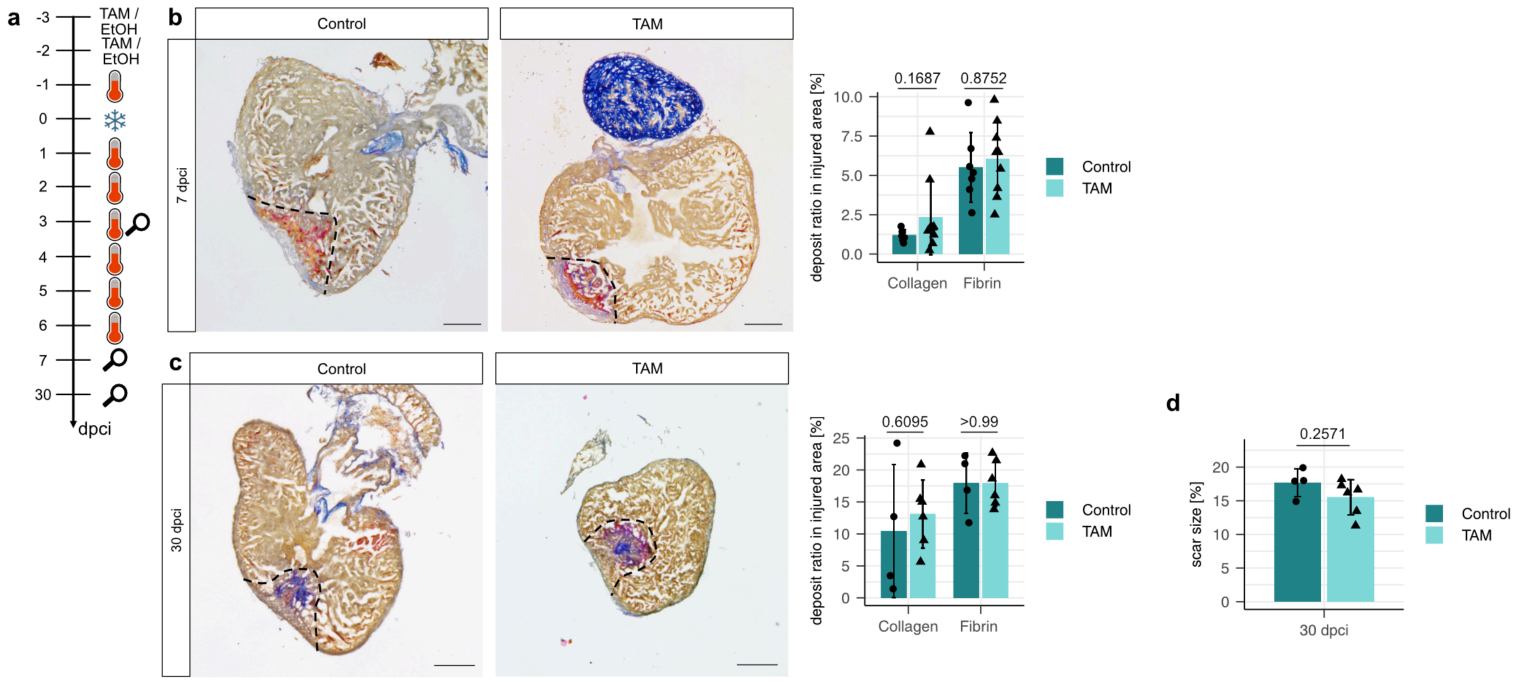

Supplementary Figure 16 Quantification of collagen and fibrin deposition, as well as total scar area in cryoinjured *TgKI(mpeg1:CreERT2);Tg(hsp:LSL-DN-MyD88)* adult zebrafish hearts. **a,** Overview of the experimental design to estimate fibrin and collagen deposition during cardiac regeneration in *TgKI(mpeg1:CreERT2);Tg(hsp:LSL-DN-MyD88)* zebrafish treated with vehicle or TAM to induce macrophage-specific overexpression of DN-MyD88. Snowflake indicates cryoinjury, thermometer indicates heat shock, and magnifying glass indicates sampling timepoint. **b-c,** Representative AFOG staining images and quantification of fibrin (red) and collagen (blue) deposition in the scar aera in 7 dpci (**b**) and 30 dpci (**c**) *TgKI(mpeg1:CreERT2);Tg(hsp:LSL-DN-MyD88)* zebrafish treated with vehicle or TAM. Black dashed lines outline the scar area. Scale bars: 200 µm. The proportions of fibrin and collagen deposition in the scar area were quantified. Dots represent individual ventricles; data shown as mean±s.d.; statistical test: Student´s t-test. **d,** Quantification of scar area in 30 dpci *TgKI(mpeg1:CreERT2);Tg(hsp:LSL-DN-MyD88)* zebrafish treated with vehicle or TAM. Dots represent individual ventricles; data shown as mean±s.d.; statistical test: Student´s t-test. **a** was partially created in BioRender. Mintcheva, J. (2026) https://BioRender.com/w02lqzq

Mathematical Supplement

Calculation of decay rates of SLAM-seq time course

To calculate decay rates, we used the pre-processed bam files of the SLAM-seq time course with MT tags. As we had observed decreased labeling rates at 24 h, likely due to reduced 4sU availability, we removed that time point from our analyses. These analyses are based on reads, not transcripts. First, we calculated the number of T and the number of T to C per read. Next, we filtered out genes with less than 25 reads. Reads with at least one T to C conversion were classified as labeled.

We assumed a steady-state system in which total gene expression levels remain constant over time. Under this assumption, the dynamics of unlabeled RNA can be described by a first-order decay model:

$$(1)\ \frac{dO}{dt} = -\gamma \cdot O$$

where:

- $O(t)$ described the unlabeled reads at time t,
- γ is the degradation rate (constant).

Solving this differential equation with the initial condition $O(0) = O_0$ gives the exponential decay of:

$$(2)\ O(t) = O_0 \cdot e^{-\gamma t}$$

The half-life τ, defined as the time at which $u(\tau) = {}^{O_0}/_{2}$, is then given by:

$$(3)\ \tau = \frac{1}{\gamma} \cdot \ln(2)$$

Assuming that total expression is constant over time (i.e., $O(t) + N(t) = cst = O_0$, with $O(t)$ the labeled reads), the ratio of labeled reads at time t is:

$$(4)\ r_{lab} = \frac{N(t)}{N(t) + O(t)} = \frac{O_0 - O(t)}{O_0} = 1 - \boldsymbol{e}^{-\boldsymbol{\gamma t}}$$

We used this final expression to fit the parameter γ for each gene. Fitting was performed using the average r_{lab} values across three biological replicates at each time point. The associated standard deviations across replicates were provided to weight the fits. Only

genes with at least two valid timepoints were considered for fitting. For each gene, we recorded the best-fit γ, the associated covariance matrix, and the coefficient of determination R^2.

Further manual inspection of the fits showed deviations from the observed plateaus (see Main Fig. 1e, *fosl2*). This observation suggests that the assumption of near-complete replacement of unlabeled RNA might not hold true for all genes. Therefore, for each gene (N = 14,553 total genes), we quantified a data-driven observed plateau by taking the last timepoint of the time window (12 h post 4sU injection). The distribution of these plateau values displays a clear multimodal structure. To characterize it, we fitted a Gaussian mixture model to the distribution, which identified three distinct components as the best fit (Supplementary Fig. 17):

- A low-plateau component centered at ~0.11, comprising 40% of genes. These genes are poorly labeled within the 12-hour window and likely reflect background signal, transcriptional inactivity, or very long half-lives.
- An intermediate component centered at ~0.39, comprising 26% of genes. These partially labeled genes may result from time-varying transcription rates $\alpha(t)$ or intermediate kinetics (see simulations below).

Genes in these first two components are not compatible with simple degradation-kinetic models and are excluded during filtering.

- A high-plateau component centered at ~0.74, comprising 34% of genes. These correspond to well-detected, short- and medium-lived transcripts that approach a steady-state plateau near the labeling efficiency/detection limit.

This distribution is consistent with previous SLAM-seq studies, where incomplete labeling and background detection have been shown to affect plateau estimation.

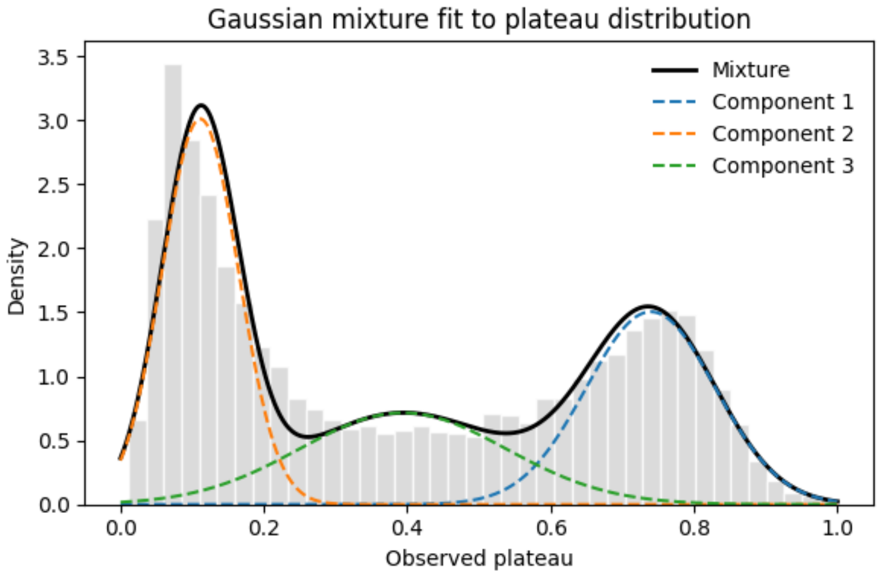

Supplementary Fig. 17 Distribution of observed labeling plateaus across genes. For each gene (N = 14,553), an observed plateau was computed as the mean labeled-RNA fraction over the last part of the labeling window (12 h labeling). A Gaussian mixture model with three components was fitted to the distribution.

As expected, our simple kinetic model (which does not include a labeling correction factor and converges to a labeling ratio of 1 at long times) fits best the high-plateau component.

To illustrate how time-varying transcription affects the approach to the labeled-RNA plateau, we simulated the dynamics of labeled L and unlabeled O RNA using a standard kinetic labeling model. One approach described for example in [1] introduces into this modeling framework a labeling-correction coefficient, denoted here as ρ. In this approach, total RNA has a synthesis rate constant α and a degradation rate constant γ. The labeled RNA has a reduced effective synthesis rate $\rho\alpha$ but the same degradation rate γ. Symmetrically, the unlabeled fraction has an effective synthesis rate $(1-\rho)\alpha$, meaning that a fraction of newly synthesized reads is assigned to the unlabeled pool.

[1] Qiu, X., Zhang, Y., Martin-Rufino, J. D., Weng, C., Hosseinzadeh, S., Yang, D., ... & Weissman, J. S. (2022). Mapping transcriptomic vector fields of single cells. *Cell*, *185*(4), 690-711.

We integrate this model under constant, increasing, and decreasing transcriptional profiles $\alpha(t)$, showing that smooth production shifts can naturally generate diverse plateau values in the labeling ratio $r_{lab}(t)$. In more detail, the system is described by:

$$\frac{dL}{dt} = \rho \cdot \alpha(t) - \gamma \cdot L(t)$$

$$\frac{dO}{dt} = (1-\rho) \cdot \alpha(t) - \gamma \cdot O(t)$$

Where

- $\alpha(t)$ is the time-dependent transcription rate,
- γ is the degradation rate,
- ρ is the labeling correction coefficient.

In the simulations shown below, we numerically integrate this system to compute $r_{lab}(t)$ under three transcriptional regimes:

(i) constant transcription:

$$\alpha(t) = cst = 1.0,$$

(ii) Transcriptional activation, modeled as a sigmoidal increase:

$$\alpha(t) = \frac{1}{\left(1+e^{-(t-t_0)/k}\right)},$$

(iii) Transcriptional repression, modeled as the corresponding sigmoidal decrease:

$$\alpha(t) = 1 - \frac{1}{\left(1+e^{-(t-t_0)/k}\right)}.$$

We used the following parameter values in the simulations presented below:

- $\rho = 0.8$ and $\gamma = \ln(2)/HL$ with $HL = 4.0\ h$.
- $t_0 = 4.0\ h$, the time at which activation or repression reaches its half-maximum (left panel), or a range of t_0 values in the delayed-activation experiments (right panel).
- $k = 0.8\ h$, the slope parameter controlling the steepness of the sigmoidal transition.

These simulations show that smooth changes in $\alpha(t)$, generate a wide range of observable plateaus in $r_{lab}(t)$, depending on the timing of transcriptional shifts relative to the degradation timescale (Supplementary Fig. 18, Supplementary Fig. 19). This demonstrates that incomplete approach to a plateau can arise from biologically plausible transcriptional dynamics rather than from model mis-specification.

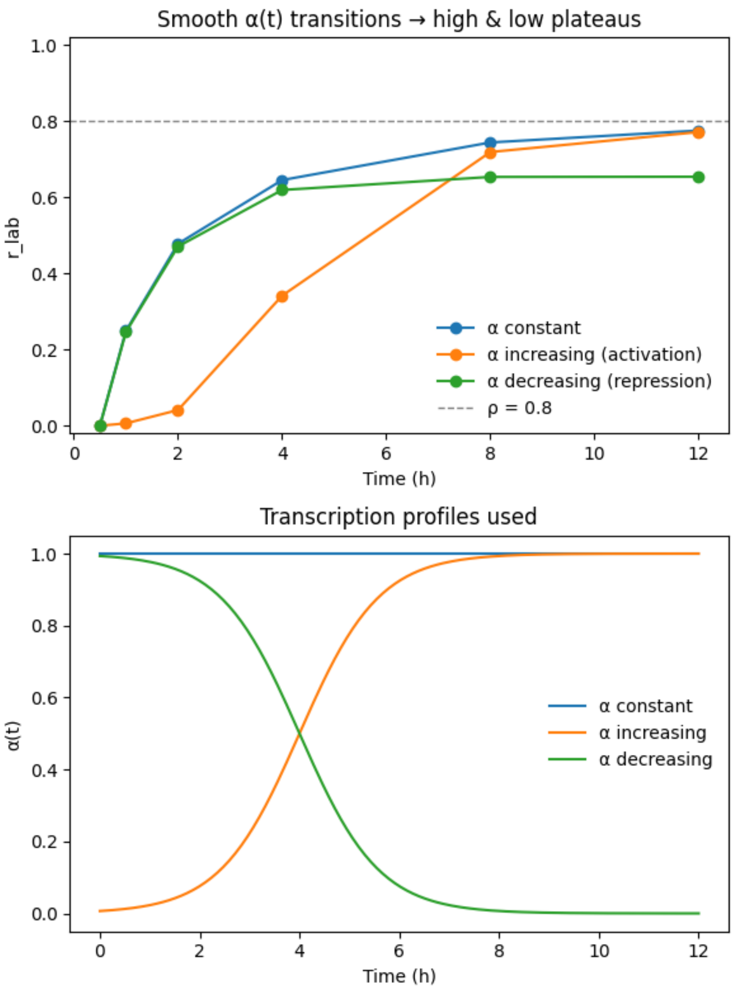

Supplementary Fig. 18 Simulated dynamics of the labeled RNA fraction under constant, increasing, and decreasing transcription rates α(t) using a kinetic labeling model with a labeling correction coefficient ρ=0.8.

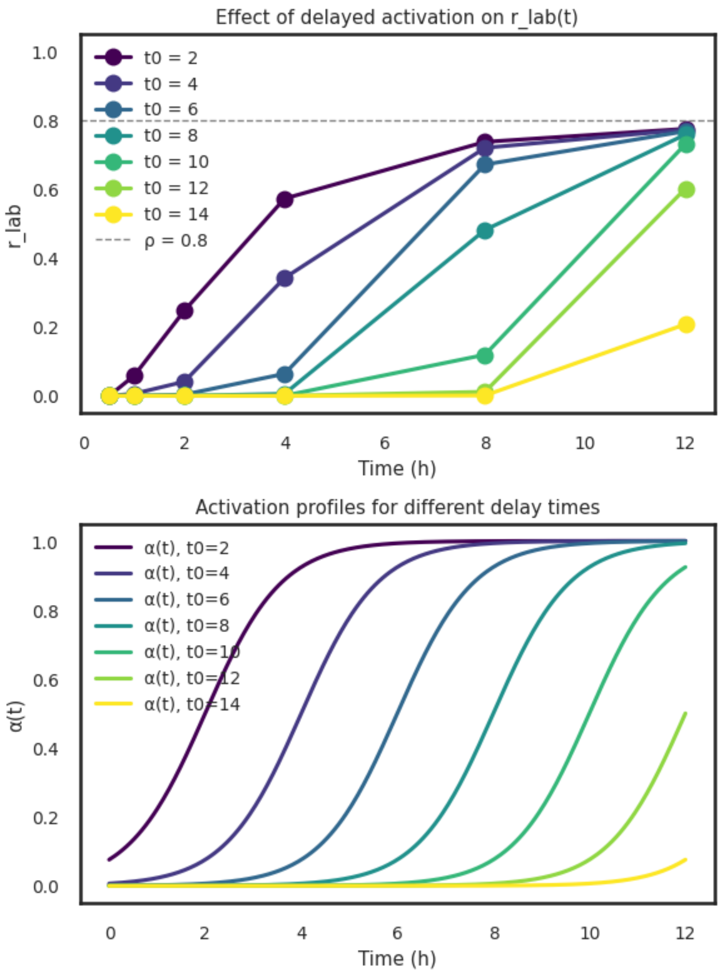

Supplementary Fig. 19 Simulated dynamics of the labeled RNA fraction for increasing delays in transcriptional activation, illustrating the dependence of the observed plateau on the timing of transcriptional changes.

To assess the impact of an imperfect labeling correction on decay rate estimation, we compare the two models describing the labeling ratio:

$$r_{lab}(\mathrm{t}) = 1 - e^{-\gamma \mathrm{t}} \text{ and } r_{lab}(\mathrm{t}) = \rho(1 - e^{-\tilde{\gamma}\mathrm{t}})$$

Then, the difference between the decay rates is given by

$$\gamma - \tilde{\gamma} = -\frac{1}{t}\ln\left(1 - r_{lab}\right) + \frac{1}{t}\left(1 - {r_{lab}}/{\rho}\right) = \frac{1}{t}\ln\left(\frac{1 - {r_{lab}}/{\rho}}{1 - r_{lab}}\right)$$

Considering the function $\mathrm{f}(r_{lab}) = \frac{1 - {r_{lab}}/{\rho}}{1 - r_{lab}}$ we note that it is strictly decreasing for $0 < \rho < 1$ and satifying $\mathrm{f}(0) = 1$. Therefore $\ln\left(\frac{1 - {r_{lab}}/{\rho}}{1 - r_{lab}}\right) < 0$ implying that for all times $\gamma - \tilde{\gamma} < 0$.Consequently, an misestimation of the labeling ratio leads to a systematic underestimation of the decay rate, affecting all genes in the same direction.

Similarly, the magnitude of the estimated decay rate can be compared by examining the derivative of the labeled fraction. In the two cases, $\dot{r_{lab}}(\mathrm{t}) = \gamma e^{-\gamma \mathrm{t}}$ and $\dot{r_{lab}}(\mathrm{t}) = \rho\tilde{\gamma} e^{-\tilde{\gamma}\mathrm{t}}$ which at initial time yield slopes γ and $\rho\tilde{\gamma}$, respectively. Since ρ is close to 1 (≈0.8 in our data), the difference in initial slopes remains limited, consistent with a moderate but systematic underestimation of the decay rate.

Injury response analysis from scSLAM-seq data

Our injury response model is based on two differential equations that describe RNA turnover in a cell, similar to the one used to calculate the decay rate in the previous section. Here, we explore the role of steady state RNA levels in the case where they change as a response to injury. The differential equation (5) encapsulates that RNA present prior to 4sU addition is degraded at rate γ, while the differential equation (6) describes the accumulation of new (labeled) RNA after 4sU addition (Supplementary Fig. 20).

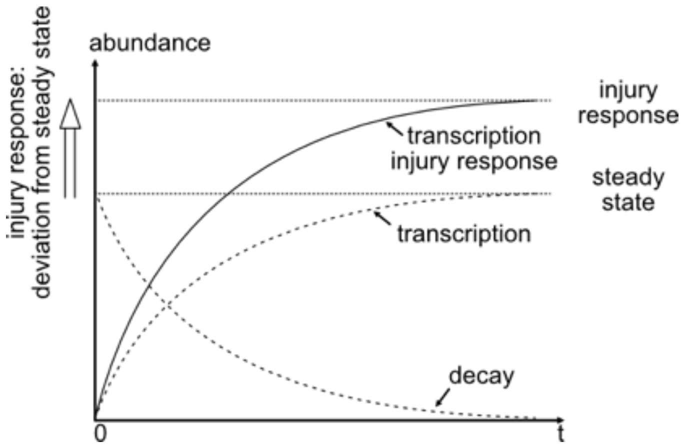

Supplementary Fig. 20 Schematic representation of decay of old (unlabeled) RNA and accumulation of new (labeled) RNA in steady state and after injury.

$$(5)\ \frac{dO}{dt} = -\gamma \cdot O \ \Rightarrow\ O(t) = O_0 \cdot e^{-\gamma t}$$

$$(6)\ \frac{dN}{dt} = k - \gamma \cdot N \ \Rightarrow\ N(t) = N_\infty \cdot (1 - e^{-\gamma t})$$

With O: old RNA (i.e. unlabeled RNA), N: new RNA (i.e. labeled RNA), t: time, k: transcription rate.

O(t=0) denotes old RNA at timepoint 0, hence RNA present prior to 4sU addition, equivalent to the steady state level before injury. N(t=inf) denotes new RNA at timepoint infinity, equivalent to the new steady state transcript level after injury (Supplementary Fig. 21). In steady state conditions the total number of transcripts, for each gene, is hence given by:

$$(7)\ steady\ state\colon O_0 = N_\infty = O(t) + N(t)$$

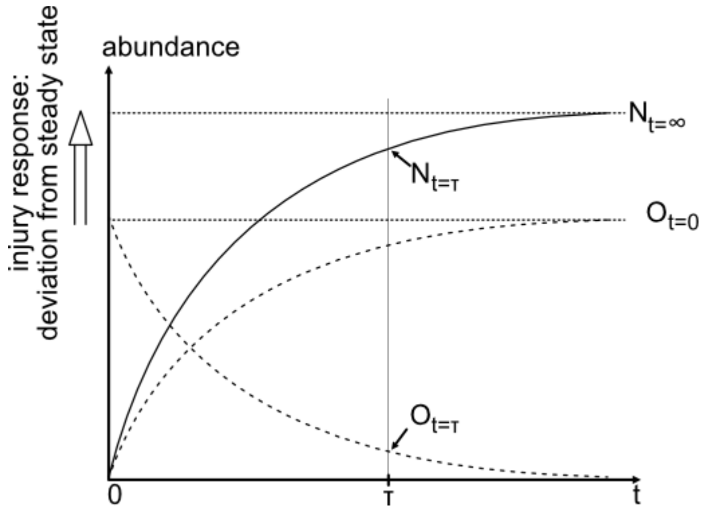

Supplementary Fig. 21 Schematic representation of transcription and decay in steady state and after injury, with abbreviations used in the formulas.

For modeling the response to injury, we acquired two scSLAM-seq datasets at t=τ with τ here being the time of labeling after 4sU injection: a perturbed sample (after injury), and a control sample (sham). 4sU was added in both samples during steady state, directly prior to injury or sham. We made the following simplifying assumptions:

- The RNA decay rates do not change as a response to injury (γ=const.)
- We assume that the sham control remains at steady state.
- In the injured sample, the steady state level of transcript abundance changes, from $O_{t=0}$ to $N_{t=¥}$ upon perturbation

We want to determine the differential expression upon injury in the new steady state (the t = ¥):

$$(8)\frac{N_\infty}{O_0} = \frac{N_\tau}{O_\tau} \cdot \frac{e^{-\gamma\tau}}{1 - e^{-\gamma\tau}}$$

Assuming steady state conditions for the sham injury, $O_0 = N_t + O_t$, we can estimate the RNA decay rates from the sham sample as:

$$(9)\ O_\tau^{sham} = O_0^{sham} \cdot e^{-\gamma\tau}$$

$$(10)\ O_\tau^{sham} = \left(O_\tau^{sham} + N_\tau^{sham}\right) \cdot e^{-\gamma\tau}$$

$$(11)\ ln\frac{O_\tau^{sham}}{N_\tau^{sham} + O_\tau^{sham}} = -\gamma\tau$$

$$(12)\ \gamma = \frac{-ln\frac{O_\tau^{sham}}{N_\tau^{sham} + O_\tau^{sham}}}{\tau}$$

Inserted into the equation of the differential expression, we obtain our injury response formula as below:

$$(13)\ injury\ response = \frac{N_\infty}{O_0} = \frac{N_\tau^{injured}}{O_\tau^{injured}} \cdot \frac{O_\tau^{sham}}{N_\tau^{sham}}$$

A closer look at the unlabeled RNA of the first replicate revealed a good, however not perfect correlation between the 6 hps and 6 hpci dataset (Supplementary Table 1).

Supplementary Table 1 Pearson correlation of 6 hps and 6 hpci unlabeled and labeled RNA.

	Pearson correlation unlabeled RNA	Pearson correlation labeled RNA
cardiomyocytes (atrium)	0.98	0.93
cardiomyocytes (ventricle)	0.98	0.95
endocardium	0.94	0.86
epicardium	0.95	0.87
fibroblasts	0.91	0.65
macrophage-like cells	0.95	0.80
neutrophils	0.92	0.76
smooth muscle cells	0.93	0.69
erythrocytes	0.99	1.00

Since the unlabeled RNA equates to steady state RNA which should be the same between samples and should not be affected by the perturbation, we assume that the non-perfect correlation corresponds to technical noise and/or pre-existing biological variation. Hence, we further simplify the equation to:

$$\boldsymbol{(14)\ injury\ response = \frac{N_\infty}{O_0} = \frac{N_\tau^{injured}}{N_\tau^{sham}}}$$

Additionally, this simplification comes with the advantage that it will not be influenced by false negative transcripts, i.e. newly transcribed RNA that is not labeled. While overall, the existence of false negative transcripts will slightly reduce the resolution, they will not falsify our analysis.

To apply equation (14) to our scSLAM-seq data, we first aggregate labeled and unlabeled raw counts each to pseudo-bulk counts per cell type. Next, we calculate the injury score as the labeled counts of the injured sample over the labeled counts of the sham sample. To account

for cell type specific difference in average cell size and overall cell numbers, we remove non-finite injury scores and normalize the injury scores by the median injury score per cell type.

While these injury scores can already be used for analyses, we wanted to take our analyses a step further and remove genes that might be dominated by noise. To estimate the noise properties of our data, we created a background model which is based on background injury scores of subsampled sham data. Here, we randomly split the sham dataset for each cell type in two and calculated background injury scores as the labeled counts of sham 1 over the labeled counts of sham 2, again normalized by the median of finite background injury scores (Supplementary Fig. 22).

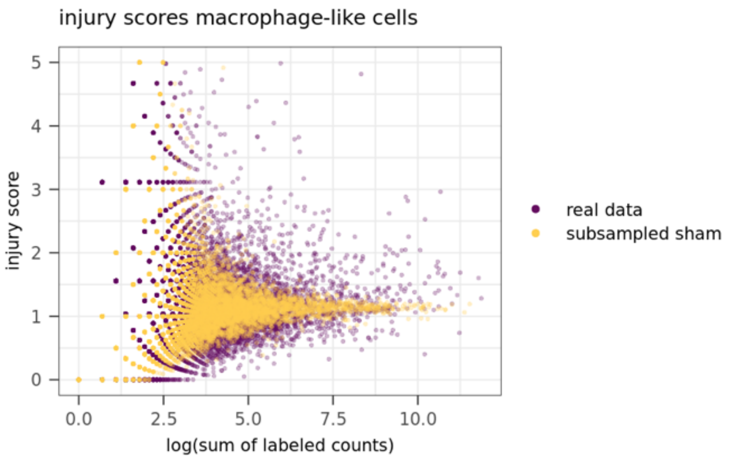

Supplementary Fig. 22 injury scores compared to background injury scores as a function of expression in macrophage-like cells.

We noted that both with the injury scores and the background injury scores, the score variability was higher in lowly expressed genes. We therefore followed the approach to apply the noise cut-off in an expression-dependent way.

To estimate the injury score variability in an expression-dependent way, we randomly split the cells of the entire sham dataset in different ratios: 50:50, 70:30, 45:55. We then calculated the pseudo-bulk ratios of labeled transcripts without discriminating between cell types. Next, we created expression bins based on the sum of labeled counts per gene and calculated the background injury score average and standard deviation and coefficient of variation of each bin while disregarding outliers above the top 99.5 percentile (Supplementary Fig. 23). We were able to observe that the coefficient of variation remained very stable across the different split ratios we tested, motivating us to use the coefficient of variation for downstream analyses (Supplementary Fig. 24).

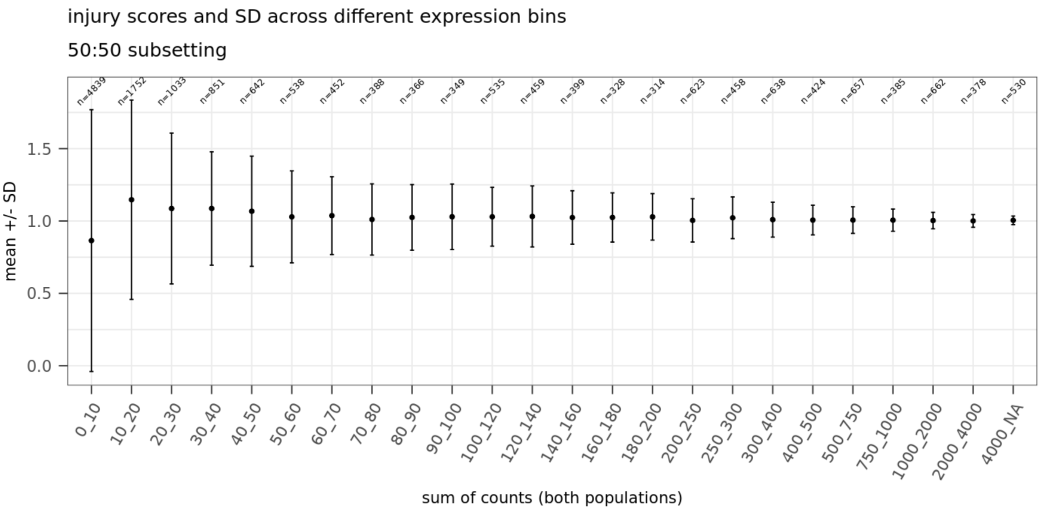

Supplementary Fig. 23 Mean and SD of background injury scores binned by expression in the 50:50 subset of the 6 hps dataset. N indicates the number of genes per bin.

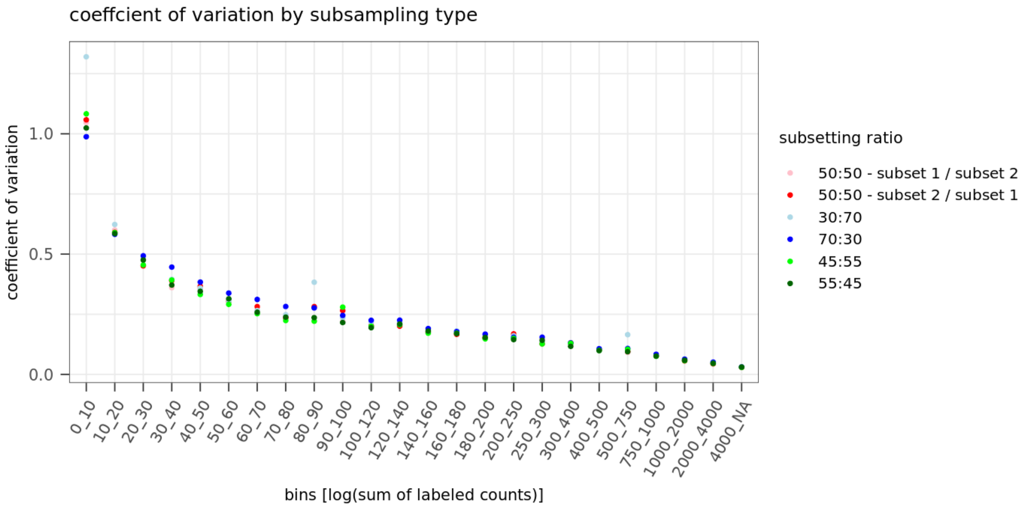

Supplementary Fig. 24 Coefficient of variation across expression bins and different subsetting ratios.

In a last step, we tied all the parts of the background model together. We overlaid the injury scores and the background injury scores, and added the expression-dependent background thresholds (Supplementary Fig. 25). Of note, our system offers high flexibility, as filtering cut-offs can be modularly combined. In our case, we required an injury score higher than 1.5, and added an overall expression filter of 10, 20 or 50 sum of labeled counts, as well as the noise threshold of the median score per expression bin plus 1, 2, or 3 standard deviations per bin, based on the coefficient of variation calculated above. Of each combination of filtering parameters, we performed GO term analyses using gprofiler2 on the genes passing those filters, using a hypergeometric test, one-sided, with g:SCS multiple testing correction. Upregulation of damage response pathways like TLR, NLR and CLR signaling pathways was observed in most filtering combinations, which validates the robustness of the analysis.

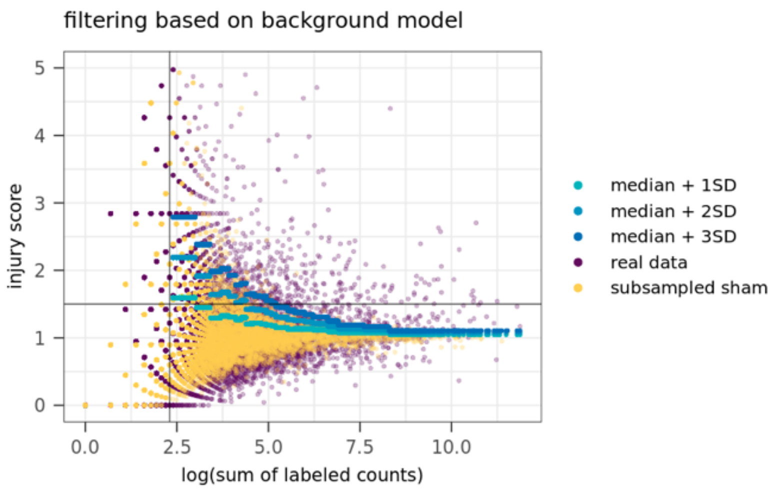

Supplementary Fig. 25 Filtering of potentially injury responsive genes in macrophage-like cells based on expression (vertical line, here: sum of labeled counts = 10), baseline injury score (horizontal line, here: injury score = 1.5) and the background model (blue dotted lines, here: median + 1/2/3 SD).

To control for our analysis method, we repeated this analysis using labeled plus unlabeled counts to create injury scores (i.e. disregarding the RNA labeling information), which allowed us to assess the power of the SLAM-seq data for determining processes that are differentially regulated upon injury.

Modeling of false negative probabilities

False negative transcripts in scSLAM-seq data are transcripts that are newly transcribed after 4sU addition but which are not detected as labeled, for example by not incorporating any 4sU molecule. A simple back-of-the-envelope calculation of the false negative probability, given the labeling probability p of an individual thymine in a newly transcribed RNA with n number of thymines can be described as

$$(15)\ false\ negative\ probability = FN = (1-p)^n$$

Among labeled transcripts in the 6 hps sample, we detect a labeling rate of 4.4% per thymine. With a Read2 sequencing read length of 202 nucleotides in our scSLAM-seq experiments among which we observe roughly 50 thymines on average, our false negative probability amounts to approximately 10.5%.

To get a more refined estimate, we can use a slightly more complex set-up. While we can only measure the observed labeling rate among labeled transcripts, we cannot detect the false negative transcripts as they have 0% labeling. The true labeling rate p, however, consists of false negative transcripts with 0% labeling rate, and labeled transcripts with a labeling rate among labeled transcripts p_{obs}. We can hence describe p and p_{obs} as:

$$(16)\ p = FN \cdot 0\% + (1-FN) \cdot p_{obs}$$

$$(17)\ p_{obs} = \frac{p}{1-(1-p)^n}$$

Given, for example, 50 thymines and an observed labeling rate of 4.4% among labeled transcripts, *p* amounts to 3.75%.

For a more generalized approach to determine the true labeling probability *p* of labeling any thymine in a transcript given uniform labeling, we calculate the chance of detecting a new transcript with *n* number of thymines in which *k* number of thymines have been labeled as a binomial formula:

$$(18)\ P(X=k) = \binom{n}{k} \cdot p^k \cdot (1-p)^{n-k}$$

To determine the labeling probability based on our experimental data, we can use the experimental ratio of detecting 2 labeled thymines over 1 labeled thymine in transcripts with the same amount of thymines, or 3 labeled thymines over 2 labeled thymines respectively:

$$(19)\ experimental\ ratio\ R\ (2\ over\ 1\ labeled\ T) = \frac{\binom{n}{2} \cdot p^2 \cdot (1-p)^{n-2}}{\binom{n}{1} \cdot p \cdot (1-p)^{n-1}} = \frac{\binom{n}{2} \cdot p}{\binom{n}{1} \cdot (1-p)}$$

$$(20)\ p = \frac{R \cdot \binom{n}{1}}{\binom{n}{2} + R \cdot \binom{n}{1}}$$

To probe our back-of-the-envelope calculation, we simulated labeling to calculate false negative probabilities. We therefore used our 6 hps scSLAM-seq dataset, and subsetted our bam file for perfect reads, i.e. reads with a Q30 quality score, a CIGAR of 202M and zero detected T to C conversions, as well as a fixed number of thymines ranging from 40 to 75 in 5-step increments. Next, we randomly substituted Ts by Cs with different probabilities, re-mapped the data, and re-ran the scSLAM-seq pipeline. We calculated the experimental ratio and false negative probabilities for fixed amount of thymines per transcripts as described above. While we observed good accordance of the false negative probabilities from the simulated data to the back-of-the-envelope calculation, we observed a deviation of the probabilities based on the 2 over 1 conversion experimental ratio of the real data (Supplementary Fig. 26). This points towards non-uniform 4sU labeling. However, this observation is not surprising given that with a single 4sU injection its availability does not remain constant over time. At the same time, we observe a good accordance in the experimental ratio of 3 over 2 conversions (Supplementary Fig. 27). This could point towards a skew to incorporate multiple 4sU molecules in a transcript, i.e. high 4sU availability or even saturation.

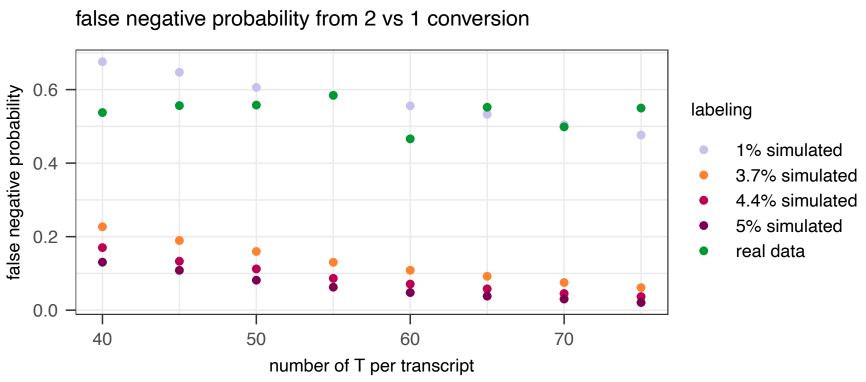

Supplementary Fig. 26 False negative probabilities of simulated and real labeling data calculated by the experimental ratio of two over one conversions

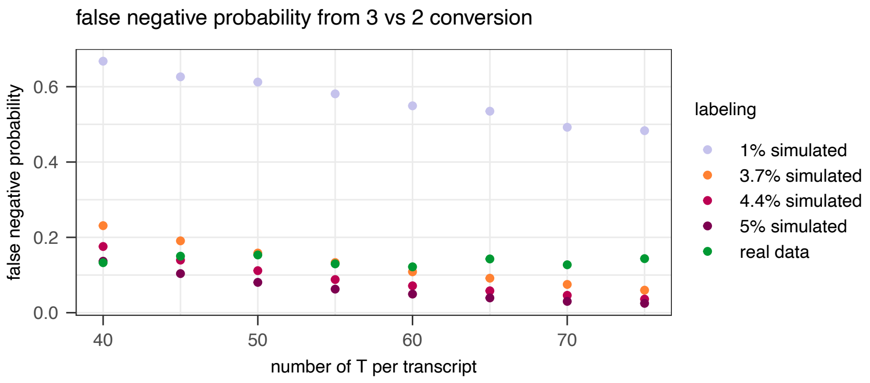

Supplementary Fig. 27 False negative probabilities of simulated and real labeling data calculated by the experimental ratio of three over two conversions
