# Supplementary material for: In vivo single-cell RNA metabolic labeling resolves early transcriptional responders in the regenerating zebrafish heart
Source: Nat Commun. 2026 May 5;17:4073. doi: 10.1038/s41467-026-72781-2 (PMC13144409; doi:10.1038/s41467-026-72781-2)
Supplement: Supplementary file 2 — Description of Additional Supplementary Files [file 41467_2026_72781_MOESM2_ESM.pdf]

Description of Additional Supplementary Files

File name: Supplementary Data 1
Description: scRNA-seq meta data.

File name: Supplementary Data 2
Description: Decay rates inferred from bulk SLAM-seq time course.

File name: Supplementary Data 3
Description: scRNA-seq and scSLAM-seq sequencing quality statistics.

File name: Supplementary Data 4
Description: Upregulated GO terms of injury responsive genes in scSLAM-seq replicate pair 1.

File name: Supplementary Data 5
Description: Seurat differential gene expression of scSLAM-seq data.

File name: Supplementary Data 6
Description: Upregulated GO terms of injury responsive genes in scSLAM-seq replicate pair 1.

File name: Supplementary Data 7
Description: Maker genes of scRNA-seq data.

File name: Supplementary Data 8
Description: Upregulated GO terms of cNMF GEPs.

File name: Supplementary Data 9
Description: Gene list of pathway layer split.

File name: Supplementary Data 10
Description: qPCR Ct values.

File name: Supplementary Data 11
Description: Primer sequences.
