# Supplementary material for: In vivo single-cell RNA metabolic labeling resolves early transcriptional responders in the regenerating zebrafish heart
Source: Nat Commun. 2026 May 5;17:4073. doi: 10.1038/s41467-026-72781-2 (PMC13144409; doi:10.1038/s41467-026-72781-2)
Supplement: Supplementary file 14 — Reporting Summary [file 41467_2026_72781_MOESM14_ESM.pdf]

nature portfolio

Corresponding author(s): Jan Philipp Junker

Last updated by author(s): Mar 31, 2026

Reporting Summary

Nature Portfolio wishes to improve the reproducibility of the work that we publish. This form provides structure for consistency and transparency in reporting. For further information on Nature Portfolio policies, see our Editorial Policies and the Editorial Policy Checklist.

Statistics

For all statistical analyses, confirm that the following items are present in the figure legend, table legend, main text, or Methods section.

n/a	Confirmed	
☐	☒	The exact sample size (*n*) for each experimental group/condition, given as a discrete number and unit of measurement
☐	☒	A statement on whether measurements were taken from distinct samples or whether the same sample was measured repeatedly
☐	☒	The statistical test(s) used AND whether they are one- or two-sided<br>*Only common tests should be described solely by name; describe more complex techniques in the Methods section.*
☒	☐	A description of all covariates tested
☐	☒	A description of any assumptions or corrections, such as tests of normality and adjustment for multiple comparisons
☐	☒	A full description of the statistical parameters including central tendency (e.g. means) or other basic estimates (e.g. regression coefficient) AND variation (e.g. standard deviation) or associated estimates of uncertainty (e.g. confidence intervals)
☐	☒	For null hypothesis testing, the test statistic (e.g. *F*, *t*, *r*) with confidence intervals, effect sizes, degrees of freedom and *P* value noted<br>*Give P values as exact values whenever suitable.*
☒	☐	For Bayesian analysis, information on the choice of priors and Markov chain Monte Carlo settings
☒	☐	For hierarchical and complex designs, identification of the appropriate level for tests and full reporting of outcomes
☐	☒	Estimates of effect sizes (e.g. Cohen's *d*, Pearson's *r*), indicating how they were calculated

Our web collection on statistics for biologists contains articles on many of the points above.

Software and code

Policy information about availability of computer code

Data collection	-Fluorescence images were acquired using ZEN 3.2 (Blue edition) software and the Nikon NIS-AR software (v.5.3).<br>-Wholemount ventricle images, AFOG staining images, and larvae images were acquired using NIS-Elements 4.30 software.<br>-RT-qPCR data were acquired using Bio-Rad CFX Manager3.1.<br>- HCR images were acquired with Leica Software.
Data analysis	Quantifications:<br>-Quantifications of immunostained data were done using the ZEN 3.2 (Blue edition) software.<br>-Quantifications for the coronary vessel coverage, fibrin deposition, and collagen deposition analyses were done using ImageJ (version 1.53c).<br><br>Statistical analysis:<br>-All statistical analyses for images and RT-qPCR were performed in GraphPad Prism (v.9).<br><br>Computational analyses<br>cNMF v1.3.4, Seurat v4.0.6, miloR 1.0.0, harmony 0.1.0, CellRanger 4.0.0, STAR v2.7.3 and v2.7.8a<br>Code used for genomics analyses, package versions, and detailed protocols are deposited on GitHub (https://github.com/jmintch/heart_regen_slam).

For manuscripts utilizing custom algorithms or software that are central to the research but not yet described in published literature, software must be made available to editors and reviewers. We strongly encourage code deposition in a community repository (e.g. GitHub). See the Nature Portfolio guidelines for submitting code & software for further information.

Data

Policy information about availability of data

All manuscripts must include a data availability statement. This statement should provide the following information, where applicable:

- Accession codes, unique identifiers, or web links for publicly available datasets
- A description of any restrictions on data availability
- For clinical datasets or third party data, please ensure that the statement adheres to our policy

We used previously published data available on Gene Expression Omnibus accession numbers GSE159032, GSE158919, GSE262247 and the NCBI SRA database Accession number PRJNA900299. Cell annotations of scRNA-seq data of Goumenaki et al. and Wei et al. were kindly provided by the authors. Data acquired in this work is deposited on Gene Expression Omnibus under the accession numbers GSE305090, GSE305094, and GSE305095.

Research involving human participants, their data, or biological material

Policy information about studies with human participants or human data. See also policy information about sex, gender (identity/presentation), and sexual orientation and race, ethnicity and racism.

Reporting on sex and gender	N/A
Reporting on race, ethnicity, or other socially relevant groupings	N/A
Population characteristics	N/A
Recruitment	N/A
Ethics oversight	N/A

Note that full information on the approval of the study protocol must also be provided in the manuscript.

Field-specific reporting

Please select the one below that is the best fit for your research. If you are not sure, read the appropriate sections before making your selection.

☒ Life sciences ☐ Behavioural & social sciences ☐ Ecological, evolutionary & environmental sciences

For a reference copy of the document with all sections, see nature.com/documents/nr-reporting-summary-flat.pdf

Life sciences study design

All studies must disclose on these points even when the disclosure is negative.

Sample size

Sample size estimation was performed to ensure adequate statistical power for detecting biologically meaningful differences in regeneration phenotypes. For comparisons between two groups, we aimed to reject the null hypothesis of no difference using a two-tailed Student´s t-test with a significance level of 0.05 and a statistical power of 80%.
For general regeneration phenotypes, assuming an expected biological difference of 10% and an empirically estimated standard deviation of 38.3, a minimum of 7 animals per group was required.
For AFOG staining analysis, fibrin and collagen deposition was quantified as a percentage of the total injured area. Assuming an expected biological difference of 5% and an empirical standard deviation of 12.83, a minimum of 8 animals per group was required.
For the qPCR analysis, assuming an expected biological difference of 10% in gene expression levels and an empirical standard deviation of 0.2, at least 4 independent biological samples per group were required.

For single-cell transcriptomics, sample size calculations were not performed but rather, performed in duplicate (scSLAM-seq) or at least triplicate for reproducibility (or higher number of replicates if cell numbers captured were low) to capture biological variability and yield robust clustering and differential expression results. Sample numbers were chosen based on prior studies using comparable single-cell sequencing approaches, for example PMID 35864193.

Data exclusions

No data were excluded from the analysis

Replication

All attempts at replication were successful

Randomization

All experiments used females and males. All animals were chosen randomly.

Blinding

Experiments and image analysis were not performed blindly.
-We needed to treat the fish with vehicle or TAM, and subsequential heat shocks, which could not be performed blindly.
-To address potential bias, we mixed the sections and performed subsequential immunostaining, imaging, and image analyses blindly. In

addition, we ensured that all researchers involved in performing experiments and data analysis underwent thorough training and adhered to standardized protocols. Quantifications were done in a consistent manner (same person, same software) every time and thus even if there is some technical bias, it is the same throughout all the samples (vehicle and TAM). This consistency helped to maintain the reliability and validity of our findings.

Reporting for specific materials, systems and methods

We require information from authors about some types of materials, experimental systems and methods used in many studies. Here, indicate whether each material, system or method listed is relevant to your study. If you are not sure if a list item applies to your research, read the appropriate section before selecting a response.

Materials & experimental systems

n/a	Involved in the study
☐	☒ Antibodies
☒	☐ Eukaryotic cell lines
☒	☐ Palaeontology and archaeology
☐	☒ Animals and other organisms
☒	☐ Clinical data
☒	☐ Dual use research of concern
☒	☐ Plants

Methods

n/a	Involved in the study
☒	☐ ChIP-seq
☒	☐ Flow cytometry
☒	☐ MRI-based neuroimaging

Antibodies

Antibodies used

Primary antibodies used were as followed:
anti-Fli1 (clone EPR4646) at 1:200 dilution (rabbit, cat. no. ab133485, Abcam);
anti-PCNA (clone PC10) at 1:200 dilution (mouse, cat. no. sc-56, Santa Cruz Biotechnology);
anti-MEF2 at 1:150 dilution (rabbit, cat. no. DZ01398, Boster Bio);
N2.261 at 1:20 dilution (mouse, developed by H. M. Blau and obtained from the Developmental Studies Hybridoma Bank);
anti-IL1 beta at 1:200 dilution (mouse, cat. no. GTX634188, Genetex);
anti-Mfap4 at 1:200 dilution (rabbit, cat. no. GTX132692, Genetex);
anti-GFP at 1:200 dilution (chicken, Aves Labs, GFP-1010);
anti-DsRed at 1:200 dilution (recognizing mCherry, Living Colors, rabbit, Takara Bio, 632496).

Secondary antibodies used (all at 1:400 dilution) were:
anti-mouse IgG (H+L) Alexa Fluor 568 (goat, cat. no. A-11004, Invitrogen);
anti-chicken IgG (H+L) Alexa Fluor 488 (goat, cat. no. A-11041, Invitrogen);
anti-rabbit IgG (H+L) Alexa Fluor 568 (goat, cat. no. A-11036, Invitrogen);
anti-rabbit IgG (H+L) Alexa Fluor 647 (goat, cat. no. A-21244, Invitrogen).

Validation

All antibodies used in this study were commercially available.

For the primary antibodies, including anti-Fli1, anti-MEF2, anti-PCNA, anti-MEF2, N2.261, anti-GFP, and anti-DsRed, were validated and reported in cardiac regeneration study in adult zebrafish following cryoinjury. (48. Goumenaki, P. et al. The innate immune regulator MyD88 dampens fibrosis during zebrafish heart regeneration. Nat Cardiovasc Res. 3(9):1158-1176 (2024).)
Details are listed as followed:

anti-Fli1, https://www.abcam.com/products/primary-antibodies/fli1-antibody-epr4646-ab133485.html
Reference: PMID: 29762122

anti-PCNA, http://www.scbt.com/p/pcna-antibody-pc10
Reference: PMID: 35264012

anti-MEF2, https://www.bosterbio.com/polyclonal-anti-mef2-antibody-dz01398-1-boster.html
Manufacturer: anti-Zebrafish Mef2 Antibody
Reference: PMID: 36513650

N2.261, https://dshb.biology.uiowa.edu/N2-261
Reference: PMID: 25557620

anti-GFP, https://www.aveslabs.com/products/anti-green-fluorescent-protein-antibody-gfp.
Manufacturer: Chickens were immunized with purified recombinant green fluorescent protein (GFP) emulsified in Freund´s adjuvant.
Reference: PMID: 34732708

anti-DsRed, https://www.takarabio.com/documents/Certificate%20of%20Analysis/632496/632496-101717.pdf
Manufacturer: This antibody recognizes DsRed-Express, DsRed-Express2, mCherry, DsRed2, E2-Crimson, tdTomato, mStrawberry, and mBanana, and both N- and C-terminal fusion proteins containing these fluorescent proteins in mammalian cell lysates.

anti-Mfap4, https://www.genetex.com/Product/Detail/Mfap4-antibody/GTX132692?

srsltid=AfmBOor3OheshlvpKDfZGr5GDAsMCpRwTDlHez7goNfdRR4mz9RF3iTj
Manufacturer: The immunogen used to generate this antibody corresponds to zebrafish Mfap4.
Reference: PMID: 41134665

anti-IL1 beta, https://www.genetex.com/Product/Detail/IL1-beta-antibody-GT289/GTX634188
Reference: PMID: 34477811

Animals and other research organisms

Policy information about studies involving animals; ARRIVE guidelines recommended for reporting animal research, and Sex and Gender in Research

Laboratory animals	Wild-type, transgenic zebrafish used in this study were from the AB strain. The following wildtype and transgenic lines were used in this study: AB, Tg(-0.8flt1:RFP)hu5333, Tg(mpeg1.1-2A-creERT2,gcry1:NLS-EGFP)as602 (this study), Tg(hsp70l:loxp-TagBFP-loxp-DN-MyD88-t2A-mCherry)bns711 (this study), Tg(mpeg1:eGFP)gl22, Tg(mfap4:mTurquoise)tud302, Tg(ubi:Cas9;U6:sgRNA-RFP)]/ Tg(Zebrabow) (PMID: 38365956).
Wild animals	N/A
Reporting on sex	All experiments used females and males. Sex was determined based on standard anatomical procedures described for the species. The study did not need sex-based analysis.
Field-collected samples	N/A
Ethics oversight	All procedures in this study adhered to the applicable ethical guidelines. Zebrafish were bred, raised and maintained following the FELASA guidelines, as well as the protocols of the Max Delbrück Center for Molecular Medicine, the Max Planck Society and the relevant local animal welfare authorities (Landesamt für Gesundheit und Soziales, Berlin, Germany, Veterinary Department of the Regional Board of Darmstadt, Darmstadt, Germany, Regierungspräsidium Karlsruhe, Germany). These practices complied with the current German legislation on animal protection and the EU Directive 2010/63/EU regarding the use of animals for scientific research. Furthermore, the standards for housing and breeding were in line with the international 'Principles of Laboratory Animal Care' (NIH Publication No. 86-23, revised 1985).

Note that full information on the approval of the study protocol must also be provided in the manuscript.

Plants

Seed stocks	N/A
Novel plant genotypes	N/A
Authentication	N/A
